# *Frankia*-Enriched Metagenomes from the Earliest Diverging Symbiotic *Frankia* Cluster: They Come in Teams

**DOI:** 10.1093/gbe/evz153

**Published:** 2019-07-19

**Authors:** Thanh Van Nguyen, Daniel Wibberg, Theoden Vigil-Stenman, Fede Berckx, Kai Battenberg, Kirill N Demchenko, Jochen Blom, Maria P Fernandez, Takashi Yamanaka, Alison M Berry, Jörn Kalinowski, Andreas Brachmann, Katharina Pawlowski

**Affiliations:** 1 Department of Ecology, Environment and Plant Sciences, Stockholm University, Sweden; 2 Center for Biotechnology (CeBiTec), Bielefeld University, Germany; 3 Department of Plant Sciences, University of California, Davis; 4 Laboratory of Cellular and Molecular Mechanisms of Plant Development, Komarov Botanical Institute, Russian Academy of Sciences, Saint Petersburg, Russia; 5 Laboratory of Molecular and Cellular Biology, All-Russia Research Institute for Agricultural Microbiology, Saint Petersburg, Russia; 6 Bioinformatics and Systems Biology, Justus Liebig University, Gießen, Germany; 7 Ecologie Microbienne, Centre National de la Recherche Scientifique UMR 5557, Université Lyon I, Villeurbanne Cedex, France; 8 Forest and Forestry Products Research Institute, Ibaraki, Japan; 9 Biocenter, Ludwig Maximilians University Munich, Planegg-Martinsried, Germany

**Keywords:** *Frankia*, metagenomes, transposases, genome instability, Nod factors, *nodU*

## Abstract

*Frankia* strains induce the formation of nitrogen-fixing nodules on roots of actinorhizal plants. Phylogenetically, *Frankia* strains can be grouped in four clusters. The earliest divergent cluster, cluster-2, has a particularly wide host range. The analysis of cluster-2 strains has been hampered by the fact that with two exceptions, they could never be cultured. In this study, 12 *Frankia-*enriched metagenomes of *Frankia* cluster-2 strains or strain assemblages were sequenced based on seven inoculum sources. Sequences obtained via DNA isolated from whole nodules were compared with those of DNA isolated from fractionated preparations enhanced in the *Frankia* symbiotic structures. The results show that cluster-2 inocula represent groups of strains, and that strains not represented in symbiotic structures, that is, unable to perform symbiotic nitrogen fixation, may still be able to colonize nodules. Transposase gene abundance was compared in the different *Frankia-*enriched metagenomes with the result that North American strains contain more transposase genes than Eurasian strains. An analysis of the evolution and distribution of the host plants indicated that bursts of transposition may have coincided with niche competition with other cluster-2 *Frankia* strains. The first genome of an inoculum from the Southern Hemisphere, obtained from nodules of *Coriaria papuana* in Papua New Guinea, represents a novel species, postulated as *Candidatus* Frankia meridionalis. All *Frankia-*enriched metagenomes obtained in this study contained homologs of the canonical *nod* genes *nodABC*; the North American genomes also contained the sulfotransferase gene *nodH*, while the genome from the Southern Hemisphere only contained *nodC* and a truncated copy of *nodB*.

## Introduction

**Fig. 1.**
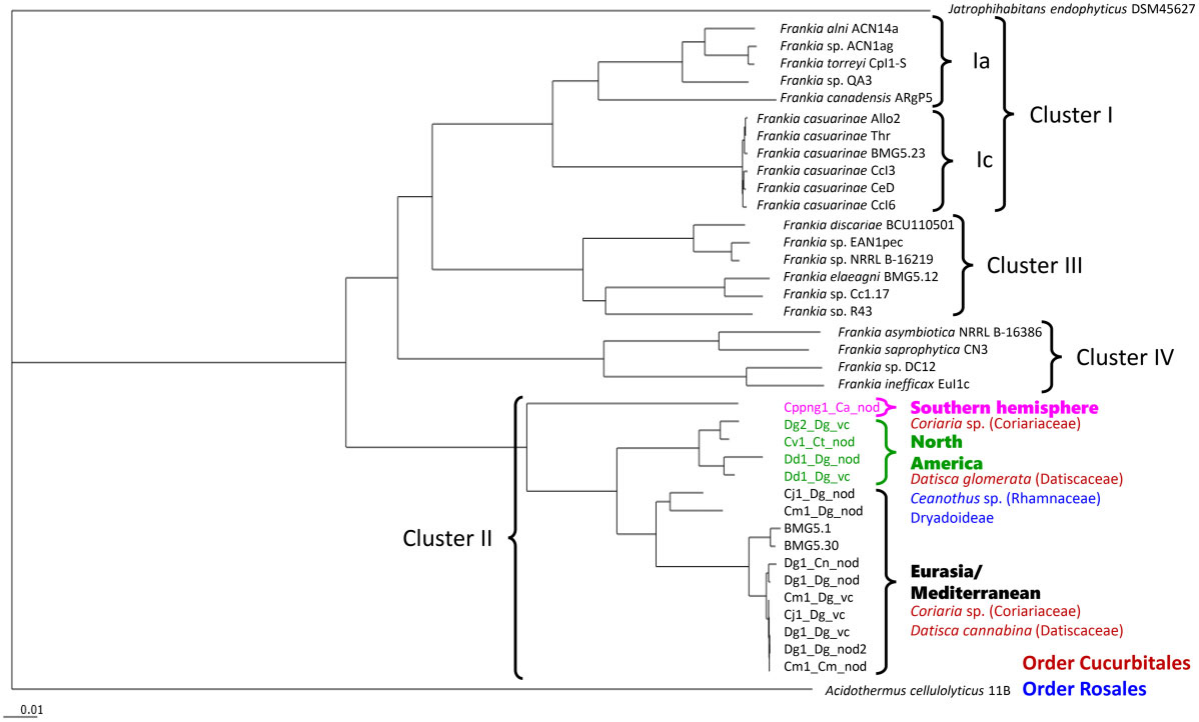
—Core genome tree of sequenced *Frankia* strains from clusters-1, -3, and -4 and of all cluster-2 (meta-)genomes available thus far (status: March 2019). The tree was calculated by means of EDGAR, deduced from concatenated core gene alignments using the neighbor-joining algorithm as implemented in the PHYLIP package PHYLIP (Felsenstein 1989). Bootstrap values were 100 for every branch (Blom et al. 2009). The scale bar denotes 0.01 substitutions. The host plant orders (red for Cucurbitales, blue for Rosales) and geographic origins (black for Eurasia, green for North America, purple for the Southern hemisphere) of the original cluster-2 inocula are color-coded. Outgroups were two actinobacterial genomes, *Acidothermus cellulolyticus 11B* (Barabote et al. 2009) and *Jatrophihabitans endophyticus* DSM45617 (GenBank accession nr. FQVU00000000.1). References for the published *Frankia* genomes are Normand et al. (2007) for ACN14a, CcI3, and EAN1pec, Sen et al. (2013) for QA3, Swanson et al. (2015) for ACN1ag, Nouioui et al. (2019) for CpI1-S, Normand et al. (2018) for ARgP5, Mansour et al. (2014) for CcI6, Oshone et al. (2016) for Allo2, Ghodhbane-Gtari et al. (2014) for BMG5.23, Hurst et al. (2014) for Thr, Ngom et al. (2016) for CeD, Wall et al. (2013) for BCU110501, Nouioui et al. (2013) for BMG5.12, Ktari et al. (2017) for NRRLB-16219, Swanson et al. (2017) for Cc1.17, Pujic et al. (2015) for R43, Ghodhbane-Gtari et al. (2013) for CN3, Tisa et al. (2015) for DC12, Nouioui, Ghodhbane-Gtari, Del Carmen Montero-Calasanz, et al. (2017) for EU1c, Nouioui, Gueddou, et al. (2017) for NRRL B-16386, Gtari et al. (2015) for BMG5.1, Gueddou et al. (2019) for BMG5.30; Persson et al. (2011) for Dg1_Dg_vc and Nguyen et al. (2016) for Dg2_Dg_vc.

Actinorhizal plants, a diverse group of dicotyledonous plants from eight families within three different orders, can form nitrogen fixing root nodules that host actinobacteria from the genus *Frankia*. Phylogenetically, *Frankia* strains can be grouped in four clusters. Three clusters represent host specificity groups (fig. 1; Pawlowski and Demchenko 2012). Cluster-1 strains nodulate actinorhizal Fagales, that is, Betulaceae; members of the Casuarinaceae except for *Gymnostoma* sp.; and members of the Myricaceae except for *Morella* species. Cluster-3 strains nodulate most actinorhizal members of the Rosales, that is, Elaeagnaceae; Rhamnaceae except for *Ceanothus* sp.; and *Gymnostoma* and *Morella*, two outlier genera of the Fagales. Strains of cluster-2, the phylogenetically basal cluster of *Frankia*, nodulate all actinorhizal Cucurbitales, that is, taxa within the Datiscaceae and Coriariaceae; and some of the Rosales, that is, the actinorhizal Rosaceae and *Ceanothus* sp. (Rhamnaceae). The fourth cluster contains noninfective or noneffective strains (Normand et al. 1996; Pozzi et al. 2018).

*Frankia* strains grow as a mycelium. In contrast with rhizobia, the nodule microsymbionts of legumes, *Frankia* strains can fix nitrogen ex planta under aerobic conditions. This is achieved by forming specialized cells, vesicles. The vesicle envelopes restrict oxygen access, thereby allowing nitrogenase function (Meesters et al. 1987; Parsons et al. 1987). In culture, vesicles are spherical to ovoid and septate, while in planta, their shape and subcellular localization is determined by the host (Newcomb and Wood 1987; Huss-Danell 1997). The fact that *Frankia* does not depend on the host in order to protect nitrogenase from oxygen is reflected in the diversity of oxygen protection systems present in different actinorhizal systems (Pawlowski and Demchenko 2012).

Since based on the latest phylogenetic studies, it represents the earliest branching cluster of *Frankia* strains, cluster-2 is of particular interest for the evolution of actinorhizal symbioses (Sen et al. 2014; Gtari et al. 2015; Persson et al. 2015; Nguyen et al. 2016; Pozzi et al. 2018). In this context, it was striking that the first cluster-2 strain to be sequenced—based on DNA isolated from symbiotic structures, namely vesicle clusters isolated from nodules—*Candidatus* Frankia datiscae Dg1, contained homologs of the canonical *nod* genes *nodABC* that encode the three enzymes responsible for synthesizing the common part of rhizobial signal factors, lipochitooligosaccharide (LCO) Nod factors, and these *nod* genes were expressed in nodules (Persson et al. 2015). Cluster-2 strains were considered unculturable until recently Gtari et al. (2015) published the isolation of an alkaliphilic strain from nodules of *Coriaria myrtifolia*, *Frankia* sp. BMG5.1, that fulfilled Koch’s postulate. The genome of this strain did not contain homologs of the canonical *nod* genes. However, in the next cluster-2 genome to be published, Dg2, a metagenome consisting of two major and one minor strain, both major strains contained the canonical *nod* genes and also a gene encoding the Nod factor sulfotransferase *nodH*, and also here, the *nod* genes were expressed in symbiosis (Nguyen et al. 2016). The configuration of the *nod* operons made clear that Dg1 and Dg2 had a common ancestor. It should be pointed out that both the Dg1 and the Dg2 genomes were isolated from nodules of the Californian species *Datisca glomerata*. However, Dg1 originated from *Coriaria nepalensis* growing in Pakistan and had been propagated in nodules of *D. glomerata* for more than a decade, while Dg2 originated from native *Ceanothus velutinus* nodules in California, and had undergone one round of propagation in nodules of greenhouse-grown plants.

These findings raise several questions. First, the fact that Dg2 represents a metagenome while Dg1 represents a single *Frankia* strain provides further evidence that in the field, cluster-2 strains exist as assemblages and that different member strains dominate in nodules of different host plants, as indicated in *Frankia* strain marker analyses in host and nonhost rhizosphere soils (Battenberg et al. 2017). The Dg1 inoculum comes from an area where both *C. nepalensis* and *Datisca cannabina* are endemic (Mirza et al. 1994; Persson et al. 2015), so it is possible that only one of its member strains was well suited for the host species *D. glomerata*. On the other hand, cluster-2 strains might occur in the field as single strains or assemblages. Second, if an assemblage of cluster-2 strains infects a host species, is the identity and contribution of the dominant strains dependent on the assemblage, as suggested by Battenberg et al. (2017), or on the host species? Can all strains that can enter the host plant differentiate nitrogen-fixing vesicles, or is vesicle formation restricted to the strain(s) best suited for the host plant species? And since the infection mechanism giving rise to root nodules in the Cucurbitales (Datiscaceae, Coriariaceae) is unknown, the question has to be asked whether individual nodules contain (an) individual strains, or whether the assemblage is more or less equally distributed over all nodules.

The third question concerns the role of the canonical *nod* genes and *nodH*. Like rhizobial Nod factors, *Frankia* signals are transduced in the host via the common symbiotic signaling pathway (CSSP), which was recruited from arbuscular mycorrhizal symbioses (Gherbi et al. 2008; Markmann et al. 2008). This pathway is commonly initiated by the binding of rhizobial LCOs to LysM receptor kinase dimers; a LysM receptor then interacts with the first component of the CSSP, SymRK (Ried et al. 2014). In view of this fact, it seemed likely that the cluster-2 *Frankia nod* genes are responsible for production of a rhizobial LCO Nod factor equivalent in *Frankia*. This is supported by the fact that *Frankia nod* genes are expressed in nodules of *D. glomerata* and of *Ceanothus thyrsiflorus* (Persson et al. 2015; Nguyen et al. 2016). However, genomes of the cultured cluster-2 strains BMG5.1 and BMG5.30 and of cluster-1 or cluster-3 *Frankia* strains, which should, and in case of *Frankia casuarinae* CcI3 have been shown to signal via the CSSP (Gherbi et al. 2008), do not contain the canonical *nod* genes (Normand et al. 2007; Gtari et al. 2015; Gueddou et al. 2019), with one exception that is likely due to lateral gene transfer (Ktari et al. 2017). Furthermore, their signal factors do not share the chemical characteristics of LCOs (Cérémonie et al. 1999; Chabaud et al. 2016). Thus, it is clear that an LCO-independent signal transduction pathway exists in the host plants of cluster-1 and cluster-3 strains and for cluster-2 hosts, at least in the *Coriaria* species that can be nodulated by BMG5.1. This pathway could be identical with the CSSP and would just require signal factor receptors with a different substrate specificity, or the direct interaction of the bacterial signal factor with SymRK. Nevertheless, the finding of conserved *nodABC* genes in strains from Pakistan and California indicated a function that was maintained under selection pressure.

In order to answer these questions, we obtained cluster-2 inocula from different places all over the world to sequence the corresponding *Frankia-*enriched metagenomes (referred to as “(meta-)genomes” in the rest of this article for simplicity, since in the majority of cases 50–70% of the sequences came from one strain). These places were Japan, where *Coriaria japonica* is the only endemic *Frankia* cluster-2 host plant; Alaska, where this role is fulfilled by *Dryas drummondii* (Rosaceae); France, where currently *C.myrtifolia* is the only host plant species, but where in the 19th century *D. cannabina* was grown to provide a dye for silk (Stenhouse 1856); and Papua New Guinea, where *Coriaria papuana* is the only host plant species. Nodules of *Cea. thyrsiflorus* harvested in California were included in the analysis to provide a second (meta-)genome from California which represents an area, where several cluster-2 host plants (*D. glomerata*, *Ceanothus* sp., *Purshia* sp., *Cercocarpus* sp., *Chamaebatia* sp.) are endemic; and to see whether the new (meta-)genome would also belong to the species *Candidatus* Frankia californiensis (Normand et al. 2017). For two of these inocula, genomes were sequenced using nodules from two different host plant species each.

## Materials and Methods

### Obtaining Field Samples

The inoculum sources are summarized in supplementary table S1, Supplementary Material online. The Cv1 inoculum originates in Sagehen Experimental Forest (near Truckee, CA) from soil and nodules collected from *Cea.velutinus* plants. The Cj1 inoculum originates from nodules harvested from *C.japonica* plants growing under pine trees in a coastal area at Tokai (Ibaraki Prefecture, Japan). A voucher sample has been deposited in the herbarium of the Swedish Museum of Natural History (S), leg. K. Pawlowski s.n. (S; Reg. No. S18-27870 (S)). The Cm1 inoculum originated from nodules harvested from a *C.myrtifolia* plant in the outskirts of Montpellier (France). Nodules from *C.papuana* Warb. harvested at Pengar River at Mt. Wilhelm, Chimbu Province, Papua New Guinea, formed the original Cppng1 inoculum. A voucher sample of the plant has been deposited in the Botanical Collection of National Herbarium Papua New Guinea Forest Research Institute, number LAE 90743. The Dd1 inoculum originates from *Dryas drummondii* nodules in the Matanuska River floodplain in Alaska (a plant voucher sample, collection number MLC2015-005, has been deposited in the herbarium of the University of Alaska at Anchorage).

### Propagation of Inocula

The isolation of vesicle clusters required 5–10 g of fresh nodules, while the isolation of total DNA from nodules required 150 mg of young nodule material (fresh or frozen). Therefore, the field samples had to be propagated before they could be used for DNA isolation. The Cv1 inoculum was propagated using *Cea.thyrsiflorus* plants that were purchased from a nursery, Corn Flower Farms (CA) in July 2012 as cuttings. For successful nodulation, the plants were repotted into new media (UC mix: perlite = 1:1) to remove any fertilizer added by the nursery. Nodulation status of each plant was checked at this point to ensure that no plants were nodulated prior to any further treatment. After 1 week, the plants were inoculated with the inoculum from Sagehen Experimental Forest. Since then the plants were maintained in a greenhouse with only deionized water and ¼-strength Hoagland solution without nitrogen (Hoagland and Arnon 1938). No exogenous nitrogen was given to the plants. The plants were kept under normal daylight except during winter when they were kept under extended artificial daylight. Nodules were harvested 8 months after inoculation. The nonlignified tips of lobes were cut off, frozen in liquid nitrogen, and kept at −80 °C until DNA isolation.

The Cj1, Cm1, and Dd1 inocula were propagated using *D.glomerata* plants; origin and conditions of cultivation and infection were as described by Nguyen et al. (2016). Cppng1 was propagated using *Coriaria arborea* plants. Seeds collected at Manganui o te Ao River near Raetihi central North Island NZ GR 39 19 04.41 S 175 13 34.95 E, elevation 305 m, were germinated for 3 weeks at 4 °C on sand wetted with tap water before transfer to the greenhouse. Further cultivation and infection conditions were the same as for *D. glomerata*. Nodules were collected 3 months after infection. For propagation of the inoculum in *C. myrtifolia* nodules, nodules of *D. glomerata* induced by the Cm1 inoculum were used to infect *C. myrtifolia* plants. Seeds of *C. myrtifolia* collected in Jijel (Algeria) were vernalized on wetted sand at 7 °C for 1 week before transfer to the greenhouse. Infection took place when the plants were ∼5 cm high. Further cultivation conditions were the same as for *D. glomerata*. Nodules were collected 8 months after infection.

A *C.nepalensis* plant was obtained from Crug Farm Plants (Caernarfon, United Kingdom). Cuttings were rooted in water, transferred to a soil/sand mixture, and infected with the Dg1 inoculum as described by Nguyen et al. (2016). Nodules were harvested 16 weeks after infection. Seeds of *Coriaria terminalis* var. *xanthocarpa* were obtained from www.plant-world-seeds.com, last accessed October 2, 2017 and germinated on a soil/sand mixture. Nodulation was performed as described for *C. myrtifolia*.

### Isolation of Bacterial Genomic DNA

Whole nodule gDNA was isolated from 400 mg of fresh root nodules using the GenElute Bacterial Genomic DNA Kit (Sigma–Aldrich, Stockholm, Sweden). The bacterial hyphae and vesicles were broken using the ultrasonic homogenizer Sonoplus HD 2070 (Bandelin Electronic, Berlin, Germany) at 30% pulsing for three times with 25 s each time.

Purification of *Frankia* vesicle clusters, and isolation of gDNA from them, was performed as described in Nguyen et al. (2016).

### Sequencing

Genomic sequencing libraries were constructed from 1 ng of gDNA with the Nextera XT DNA Sample Preparation Kit (Illumina) according to the manufacturer’s protocol. The libraries were quality controlled by analysis on an Agilent 2000 Bioanalyzer with Agilent High Sensitivity DNA Kit (Agilent Technologies) for fragment sizes of ∼200–500 bp. Sequencing on a MiSeq sequencer (Illumina; 2×250 bp paired-end sequencing, v3 chemistry) was performed in the Genomics Service Unit (LMU Biocenter, Martinsried, Germany). Raw reads were trimmed for quality (>Q20) and adapter sequences.

In order to avoid problems with GC-rich regions, for the genome sequences of Cppng1_Ca_nod and Dd1_Dg_vc whole-genome-shotgun PCR-free libraries (Nextera DNA Sample Prep Kit; Illumina, Munich, Germany) were generated based on the manufacturer’s protocol and sequenced on the MiSeq platform at the Center for Biotechnology (CeBiTec, Bielefeld University, Bielefeld, Germany).

### Genome Reconstruction and Comparative Genome Analyses

After sequencing and processing of the *Frankia* data sets, de novo assemblies were performed using the gsAssembler 2.8 (Roche) with default settings. In a next step, all raw reads were aligned to the corresponding assembled (meta-)genome contigs using Bowtie 2 (v2.2.4; Langmead and Salzberg 2012). By means of SAMtools (v1.0; Li et al. 2009), the SAM file was converted to BAM, the alignment file was sorted, and read mapping statistics were calculated. To divide the (meta-)genome contigs into genome bins, MetaBAT (v0.21.3; Kang et al. 2015) was applied with default settings. Resulting bins that represented the *Frankia* genomes were used as reference to reconstruct the corresponding *Frankia* genome. Raw reads were exported by means of mapping to the bins and reassembled using again the gsAssembler 2.8 (Roche) with default settings. Completeness, contamination, and strain heterogeneity were estimated with BUSCO (v2.0; Simã*o* et al. 2015), using the bacterial-specific single-copy marker genes database (odb9). Data were plotted by BUSCO plot (v2). For Cj1_Dg_nod and Cm1_Dg_nod, to estimate the relationship between the detected strains and the corresponding vc-strains, all binned contigs of were compared with the final draft genome of their vc-variants by applying BLASTN (threshold >1×10^−20^; Altschul et al. 1997).

Read mapping and SNP calling were performed as recently described (Rupp et al. 2015). Briefly, reads were mapped to the final draft genome sequences of Dg1_Cn_nod and Dg1_Dg_nod with Bowtie2 (Langmead and Salzberg 2012). The Genome Analysis Toolkit (GATK) IndelRealigner algorithm (McKenna et al. 2010) was applied for indel realignment, whereas SNPs were called using the GATK HaplotypeCaller algorithm (McKenna et al. 2010). Identified SNPs were exemplarily checked with ReadXplorer 2.2.3. (Hilker et al. 2014, 2016).

For the annotation of the genomes, Prokka (Seemann 2014) and GenDB (Meyer et al. 2003) were applied. Draft genome sequences were deposited at the EMBL/GenBank/DDBJ databases in BioProjects PRJEB19438–49 (for details, see supplementary table S2, Supplementary Material online).

Completeness of the reconstructed draft genomes and bins were estimated by calculating the content of bacterial BUSCOs (e-value: 0.001, data set v.3.0.2) (Waterhouse et al. 2018). The results are shown in supplementary table S3, Supplementary Material online.

The reconstructed and annotated *Frankia* (meta-)genomes were used for comparative genome analyses. Comparative analyses between the different available *Frankia* (meta-)genomes were accomplished using the comparative genomics program EDGAR 2.0 (Blom et al. 2009, 2016). Comparative analyses comprised identification of orthologous genes and classification of genes as core genes or singletons as well as the creation of phylogenetic tree based on the core genome.

### Localization of DNA in *Ceanothus thyrsiflorus* Nodules

Nodules of *Cea. thyrsiflorus* were harvested 8 months after infection with Cv1. Several nonlignified tips of nodule lobes were cut off and fixed in 3% paraformaldehyde, 0.1% Tween-20, 0.1% Triton X-100 in 10 mM phosphate buffer pH 7.2 overnight before being washed and dehydrated in a graded EtOH series until 70% EtOH and stored at room temperature. Later, they were rehydrated in a graded EtOH series and embedded in 2% agarose (SeaKem LE agarose, Cambrex, Karlskoga, Sweden). Longitudinal sections (45 µm) were prepared on an vibrating blade microtome HM 650 V (Microm, Walldorf, Germany), stained with 0.001% 4′,6-diamidino-2-phenylindole (DAPI) for 30 min and analyzed under a confocal laser scanning microscope LSM 780 (Carl Zeiss, Jena, Germany).

### Identifying Transposases and Inverted Repeats

To identify transposases in the *Frankia* genomes, the draft genomes (see table 1) were subjected to multiple BLASTX searches (Ye et al. 2006) against a database of transposase amino acid sequences. Each (meta-)genome was searched against 5,180 transposase ORF amino acid sequences, mostly from the ISfinder web site (Siguier et al. 2006) as of May 2015.

**Table 1:** List of (Meta-)Genomes from Nodules Induced by *Frankia* Cluster-2 Inocula

Inoculum	Plant Species of Origin	Plant Species for Propagation of Inoculum	Isolated from Whole Nodules (nod) or Vesicle Clusters (vc)	Metagenome Name	Number of Strains	Number of Major Strains	Ratio of Strain Contributions	Genome Size (Mb)	% GC	Sequence Similarity between Major Strains (16S)	*nod* Genes
Cj1	*Coriaria japonica*	*Datisca glomerata* ^a^	nod	Cj1_Dg_nod	2	2	59/41	8.526	68.24	86–92%	*nodABCnltIJ*
	* *	*Datisca glomerata*	vc	Cj1_Dg_vc	1	1	n.a.	5.044	69.94	n.a.	*nodABCnltIJ*
Cm1	*Coriaria myrtifolia*	*Coriaria myrtifolia*	nod	Cm1_Cm_nod	1	1	n.a.	4.953	70.22	n.a.	*nodABCnltIJ*
	* *	*Datisca glomerata* ^a^	nod	Cm1_Dg_nod	2	2	72/28	9.779	69.28	86–92%	*nodABCnltIJ*
	* *	*Datisca glomerata*	vc	Cm1_Dg_vc	1	1	n.a.	5.069	69.82	n.a.	*nodABCnltIJ*
Cv1	*Ceanothus velutinus*	*Ceanothus thyrsiflorus* ^b^	nod	Cv1_Ct_nod	2	1	93/7	5.499	68.07	n.a.	*nodABCHnltIJ*
Cppng1	*Coriaria papuana*	*Coriaria arborea*	nod	Crpng1_Ca_nod	1	1	n.a.	5.046	67.50	n.a.	*nodB2'CnltIJU*
Dd1	*Dryas drummondii*	*Datisca glomerata*	nod	Dd1_Dg_nod	1	1	n.a.	5.435	67.80	n.a.	*nodABCHnltIJ*
		*Datisca glomerata*	vc	Dd1_Dg_vc	1	1	n.a.	5.573	67.97	n.a.	*nodABCHnltIJ*
Dg1	*Coriaria nepalensis*	*Datisca glomerata* ^c^	vc	Dg1_Dg_vc	1	1	n.a.	5.323	70.04	n.a.	*nodABCnltIJ*
	* *	*Datisca glomerata*	nod	Dg1_Dg_nod1	1	1	n.a.	4.888	70.14	n.a.	*nodABCnltIJ*
	* *	*Datisca glomerata* ^d^	nod	Dg1_Dg_nod2	>1	n.d.	n.d.	5.548	69.32	n.d.	*nodABCnltIJ*
	* *	*Coriaria nepalensis* ^d^	nod	Dg1_Cn_nod	>1	n.d.	n.d.	5.191	69.55	n.d.	*nodABCnltIJ*
Dg2	*Datisca glomerata*	*Datisca glomerata* ^e^	vc	Dg2_Dg_vc	3	2	60/40/1	5.929	67.90	99%	*nodABCHnltIJ*

^a^ For numbers and relatedness of strains in Cj1_Dg_nod and Cm1_Dg_nod, see supplementary figure S2, Supplementary Material online.

^b^ Relatedness of strains in Cv1_Ct_nod was not quantified.

^c^ Maintained in *D. glomerata* over a period of 10 years before isolation of vesicle clusters for sequencing; Persson et al. (2015).

^d^ Dg1_Dg_nod2 and Dg1_Cn_nod contain several very similar strains (supplementary fig. S3, Supplementary Material online) the exact relatedness of which could not be quantified.

^e^ Nguyen et al. (2016).

n.a., not applicable; n.d., not determined.

To parse and analyze search results with accuracy and repeatability, a multistep process was employed: First, all areas of a (meta-)genome that contained one or more transposase hits (e-value cutoff 10^−4^) were designated as “footprints.” Then, the footprints were subjected to BLASTX searches (e-value cutoff 10^−4^) to identify the transposase occupying it. In the case of several BLASTX hits, the transposase hit with the highest score was chosen. If this hit did not cover the entire footprint sequence, the remainders were searched until the footprint had transposases designated to its entire sequence. Then, since transposases were occasionally identified as several fragments, an in-house script was used to join these fragments into single units. The criteria for joining two fragments were 1) that the fragments should be within 1 kb of each other; 2) that the fragments should be hits to transposases on the same strand; and 3) that the fragments should be hits to consecutive parts (±50 amino acids) of transposases.

A fraction of transposase value was computed for each transposase hit to evaluate its completeness. This value was calculated as the number of nucleotides covered by the transposase hit divided by the full nucleotide length of that transposase. Joined hits were considered as one transposase for these purposes, and the *fraction of transposase* value was computed as the sum of their coverages divided by the shortest of the originating transposases. For statistical analysis, Wilcoxson rank sum test was performed with the wilcox.test function of the R program for statistical computing (R Core Team 2019).

Scripts and data used, as well as GenBank files with annotated transposase hits for the investigated *Frankia* (meta-)genomes, are available in supplementary file S1, Supplementary Material online.

Inverted repeats were identified with the “Find repeats” function in Unipro UGENE v. 1.18.0 using the following settings: Window size = 12, Minimum identity per window = 92%, min and max distance between repeats = 0, 11,000 bp and the “Search for inverted repeats” option checked (Okonechnikov et al. 2012).

### Nucleotide Alignments

Nucleotide alignments were performed in Geneious 7.1.7 (Kearse et al. 2012) using the Geneious algorithm, “global alignment with free end gaps,” 65% similarity cost matrix, open penalty 12, extension penalty 3, automatic sequence direction.

### Protein Phylogeny

To investigate the evolutionary history of *Frankia* sp. Cppng1 putative *nodU* gene (*cmcH*), a phylogenetic analysis was conducted in combination with three sets of sequences. First, known NodU sequences of 11 rhizobial genera were collected from GenBank. Up to three sequences were collected from each genus from different species or strains. Species with their genome sequences available in Integrated Microbial Genomes (IMG) (Markowitz et al. 2014) were preferred over species without. Next, for each species with their genome available in IMG, by using its NodU sequence as a query for BLASTP (Altschul et al. 1990) against its genome, all NodU-like (but not NodU) carbamoyl transferases with similarity score better than 1e^−20^ were collected. Finally, by using the putative NodU sequence from *Frankia* sp. Cppng1 as a query for BLASTP against NCBI nonredundant database, carbamoyl transferases highly similar to *Frankia* sp. Cppng1 putative NodU (1e^−150^ or better, 45% amino acid identity or better) were collected from a phylogenetically diverse group of Actinobacteria (Sen et al. 2014).

These sequences were first aligned using MAFFT v7.272 (Katoh and Standley 2013) with accuracy-oriented alignment parameters (–localpair –retree 2 –maxiterate 1,000). Then the best substitution model for this multiple sequence alignment was calculated using ProtTest3 (Darriba et al. 2011). PROTTEST3 predicted the best substitution model to be LG with invariable sites, with gamma distribution, and with empirical base frequencies (LG+I + G+F) based on Bayesian information criterion (BIC), Akaike information criterion (AIC), and corrected Akaike information criterion (cAIC). These model parameters were transferred to RAxML v8.2.8 which was used to reconstruct the phylogeny based on maximum likelihood (Stamatakis 2014). Four parallel runs were conducted and only the best one was kept. 100 bootstrap replicates were conducted.

### RNA Isolation, DNase Digestion, and RT-qPCR Analyses

Nodules were ground in liquid nitrogen with mortar and pestle. The *Frankia* vesicles were broken using the ultrasonic homogenizer Sonoplus HD 2070 (Bandelin Electronic, Berlin, Germany) at 90% amplitude and 30% pulsing three times for 25 s each. RNA samples were isolated according to the protocol of the Spectrum Plant Total RNA kit from Sigma–Aldrich (Stockholm, Sweden) with on-column gDNA digestion by the RNase-Free DNase Set (Qiagen, Minden, Germany).

Three biological samples of 100 mg each were analyzed for each type of nodule (100 mg were represented by one to three nodules). The integrity of RNA samples were analyzed by the Agilent 2100 Bioanalyzer system (Agilent Technologies). All RNA samples that were chosen for further analyses had RNA Integrity Number (RIN) values >8.5. Reverse transcription were performed using the SuperScript IV First Strand Synthesis System (Thermo Fisher Scientific). For each gene, primers were designed based on the conserved regions of sequences from the Dg1 genome and the metagenome Dg2, except for *nodU*, which was based on the Cppng1 metagenome sequence, using Gemi (Sobhy and Colson 2012). Each qPCR reaction contained 1× Maxima SYBR Green qPCR Master Mix (ThermoFisher Scientific), 300 nM of each primer, and 4 ng of cDNA in a reaction volume of 10 µl. The conditions of qPCR was as followed: after 10 min at 95 °C were 40 cycles of 15 s at 95 °C, 30 s at 60 °C, and 30 s at 72 °C, followed by melt curve program (15 s at 95 °C, 15 s at 60 °C, and 15 s at 95 °C). Gene expression values were normalized against *infC*, the gene encoding translation initiation factor IF3. Data preprocessing and normalization were performed using GenEX (MultiD Analyses, Sweden). Primer sequences are given in supplementary table S4, Supplementary Material online.

## Results and Discussion

### Sequencing of 12 Different (Meta-)Genomes Based on Five Different Inocula

The inocula from nodules of *C.japonica*, *Dryas drummondii*, and *C.myrtifolia* were used to nodulate *D.glomerata* and (meta-)genomes were sequenced from these *D. glomerata* nodules. They were sequenced in two approaches: first, from vesicle clusters isolated from nodules and second, by direct sequencing of total DNA isolated from nodules and bioinformatic removal of non-*Frankia* sequences. Some of the *D. glomerata* nodules were used to infect other host plant species, and the resulting nodules were used for direct genome sequencing. All (meta-)genome sequences are described in table 1. To distinguish between the different (meta-)genome sequences derived from one inoculum, the following nomenclature was developed: name of inoculum, followed by the initials of the host plant species from which the metagenome was isolated, followed by either “vc” for (meta-)genomes obtained from DNA from isolated vesicle clusters or “nod” for (meta-)genomes obtained by direct sequencing of DNA isolated from whole nodules. Thus, for example, the inoculum Cm1 from *C. myrtifolia* nodules collected in Montpellier (France) gave rise to three different (meta-)genomes, Cm1_Dg_vc, Cm1_Dg_nod, and Cm1_Cm_nod. The previously published genome sequence Dg1 (Persson et al. 2015) becomes Dg1_Dg_vc while Dg2 (Nguyen et al. 2016) becomes Dg2_Dg_vc. Cv1_Ct_nod was directly sequenced from *Cea.thyrsiflorus* nodules harvested in California. Three _nod versions were sequenced of the previously published *Candidatus* Frankia datisca Dg1 inoculum (Persson et al. 2011, 2015), Dg1_Dg_nod1 and Dg1_Dg_nod2 from nodules of *D. glomerata* and Dg1_Cn_nod from nodules of *C.nepalensis* induced by Dg1.

*Datisca* *glomerata* was used for propagation of the different inocula because for this species, the procedure for isolating vesicle clusters from nodules was well established (Persson et al. 2015; Nguyen et al. 2016). Attempts to obtain *Frankia* genomic DNA from nodules of *Cea. thyrsiflorus* had been successful when nodules were used as starting material. However, it was never possible to obtain significant amounts of DNA isolated from vesicle clusters isolated from *Cea. thyrsiflorus* nodules. This could not be due to the absence of DNA from vesicles in *Cea. thyrsiflorus* nodules since they showed strong staining with DAPI (supplementary fig. S1, Supplementary Material online). For the only inoculum from the Southern Hemisphere, Cppng1, *C.arborea* had to be used for propagation since the inoculum did not nodulate either *D. glomerata* or *D. cannabina*. The features of all (meta-)genomes obtained in the course of this study, together with the already published (meta-)genomes of *Frankia* cluster-2 strains or strain assemblages, are summarized in table 1.

Thus, in this study, altogether 14 (meta-)genomes were compared that originated in seven different *Frankia* cluster-2 inocula from four continents (table 1 and supplementary table S1, Supplementary Material online). Five of these (meta-)genomes were sequenced from DNA isolated from symbiotic structures, vesicle clusters (Dg1_Dg_vc, Dg2_Dg_vc, Dd1_Dg_vc, Cm1_Dg_vc, Cj1_Dg_vc; Persson et al. 2015; Nguyen et al. 2016; this study) while the other nine were sequenced based on DNA isolated from whole nodules and thus could include strains that were not able to form vesicles. Two different DNA isolations from Dg1-induced nodules were used for sequencing; one of them (Dg1_Dg_nod1) represented one strain and the other one (Dg1_Dg_nod2) represented three strains (table 1). In short, a single inoculum can lead to considerable variety in nodule occupancy.

Based on the fully assembled chromosome of Dg1 (Persson et al. 2015) and the genomes of *Frankia coriariae* BMG5.1 (Gtari et al. 2015; Nouioui, Ghodhbane-Gtari, Rohde, et al. 2017) and the (meta-)genomes containing one dominant strain (table 1), the average genome size of a cluster-2 strain is 5.2 ± 0.3 Mb. The metagenomes Cm1_Dg_nod and Cj1_Dg_nod consist of two strains each, and the genomes of these two strains are rather diverse (86–92% DNA sequence identity; supplementary fig. S2, Supplementary Material online). This was also reflected by the apparent genome size. If the major strains forming a metagenome were rather similar (99% identity of 16S rDNAs for the two dominant members of Dg2_Dg_vc; Nguyen et al. 2016), most sequence differences would appear as SNPs in the assembled metagenome, and the apparent genome size (5.9 Mb for Dg2_Dg_vc) would not be much larger than the average of metagenomes with only one dominant strain. If, however, the major strains showed strong sequence differences as in the cases of Cm1_Dg_nod and Cj1_Dg_nod, most genome regions would be assembled independently for both strains, leading to an apparent genome size of 8.5–9.8 Mb, that is, nearly double the size of metagenomes with only one dominant strain. However, even in those cases it was not possible to separate the genomes via binning, because only few contigs were strain-specific; in most cases, the divergent regions were interspersed with conserved ones.

### A Phylogenetic Tree Based on the Entire Core Genome Shows That (Meta-)Genomes Coming from the Same Inoculum Can Display Strong Differences

All *Frankia* cluster-2 (meta-)genomes listed in table 1 and several published genomes from *Frankia* clusters-1, -3, and -4 were used to reconstruct a phylogenetic tree, build out of a core of 348 genes per genome, using EDGAR 2.0 (Blom et al. 2009, 2016) which is depicted in figure 1. In this tree, Cppng1_Ca_nod is sister to all other *Frankia* cluster-2 genomes. This placement is consistent with the fact that in previous phylogenies that contained sequences from strains from the Southern Hemisphere, these strains always occupied the basal position in cluster-2 (Benson et al. 1996; Clawson et al. 2004; Nouioui et al. 2014; Nguyen et al. 2016). Furthermore, a clear separation can be seen between the Eurasian and the North American cluster-2 (meta-)genomes.

At least one each of the (meta-)genomes from the inocula Dg1, Dg2 (Nguyen et al. 2016), Cj1, Cm1, and Cv1 represented more than one cluster-2 *Frankia* strain. The only exception in this regard was the Alaskan inoculum Dd1, and this might simply be due to the fact that the other strain(s) were underrepresented compared with the major strains as was the case for Dg1 (compare Dg1_Dg_vc; Persson et al. 2015, with Dg1_Dg_nod1 and Dg1_Dg_nod2 from this study). Thus, the data obtained in this study imply that cluster-2 *Frankia* strains often appear in groups, a fact that in combination with our inability to culture most of them may be responsible for their wide apparent host range in that 1) an inoculum can be made up from strains with different host specificity and 2) strains that do not nodulate the host plant used to prepare the inoculum might be carried over because they might grow on the nodule surface. (Meta-)genomes sequenced by Nguyen et al. (2016) and in this study contained maximally three different strains; however *Frankia* cluster-2 inocula are very likely to contain more than two to three strains since the (meta-)genomes shown here can only encompass strains that actually colonize nodules of a particular host plant. It is important to note that the fact that cluster-2 inocula represent assemblages makes cross-inoculation studies hard to interprete. Not only can different strains be responsible for the nodulation of different host plants but also negative results can be open to doubt as well as it is not clear whether the composition of an assemblage is stable during the propagation in one host plant. Strains able to nodulate other host plants might get lost.

When comparing the different (meta-)genomes derived from the same inoculum, two factors have to be taken into account. First, a _vc genome is based mostly on symbiotic structures, vesicle clusters. Since in planta, vesicle clusters are embedded in a pectin-rich matrix (Liu and Berry 1991), sequences from (a) *Frankia* strain(s) that do(es) not form vesicles in these nodules could still turn up, but only as minor contaminations. Second, the isolation of vesicle clusters that leads to _vc (meta-)genomes requires up to 10 g of nodule material, while the isolation of total DNA from nodules that leads to _nod (meta-)genomes requires only 400 mg, that is, two to five nodules. Thus, _nod (meta-)genomes will not necessarily be dominated by the strains that can form vesicles—that is, provide the plant with fixed nitrogen—and since they represent only a few nodules, they might be dominated by strains that represent minor members of the inoculum. This is underscored by the two different _nod (meta-)genomes from Dg1_Dg: Dg1_Dg_nod1 represents a single strain while Dg1_Dg_nod2 represents several closely related strains (supplementary fig. S3, Supplementary Material online). Furthermore, a comparison of Cm1_Dg_vc versus Cm1_Dg_nod and of Cj1_Dg_vc versus Cj1_Dg_nod shows that inocula can contain strains that differ dramatically with only 86–92% DNA sequence identity (table 1 and supplementary fig. S2, Supplementary Material online).

The phylogenetic tree (fig. 1) made clear that the strains represented by DNA isolated from vesicle clusters of all Eurasian inocula (Cj1_Dg_vc, Cm1_Dg_vc, Dg1_Dg_vc), as well as the dominant strains in the Eurasian _nod metagenomes (Cm1_Dg_nod and Cj1_Dg_nod; 60–70% of total *Frankia* DNA from nodules; table 1), showed striking sequence conservation (99–100% sequence identity; supplementary fig. S2, Supplementary Material online). This high similarity among the _vc versions of Eurasian cluster-2 genomes might be ascribed to the fact that they all were propagated in nodules of the same plant species, *D.glomerata*. It remains to be examined whether the _vc versions of the same inocula would change, when other host plants are used for propagation. At any rate, this result showed that strain assemblages from France, Pakistan, and Japan contained one strain the genome of which was very highly conserved.

A comparison of the different versions of (meta-)genomes from a single inoculum showed that the _nod metagenomes Dd1_Dg_nod, Dg1_Dg_nod1, Dg1_Dg_nod2, and Dg1_Cn_nod represented the strain that dominated the corresponding vesicle cluster-based genomes (Dd1_Dg_vc and Dg1_Dg_vc, respectively), or (a) very similar strain(s). In case of the Dg1 inoculum, this may be due to the fact that the inoculum goes back to *C. nepalensis* in Pakistan and was propagated in nodules of the Californian species *D. glomerata* in greenhouses for two decades (Persson et al. 2015) which may have led to the loss of strains from the assemblage.

However, two _nod versions, Cm1_Dg_nod and Cj1_Dg_nod, also contained strains of their respective assemblages that were not strongly represented in the corresponding _vc versions. These two outlier strains present in Cj1_Dg_nod (from Japan) and Cm1_Dg_nod (from France) were quite different from the strain in the respective vesicle clusters (86–92% sequence identity overall; supplementary fig. S2, Supplementary Material online); they were more similar to each other than to the genomes of the strains that dominated the _vc version of their inocula (fig. 1 and supplementary fig. S2, Supplementary Material online).

The fact that a group of a few individual nodules can contain a strain (at 30–40% of total *Frankia* DNA from nodules) that does not seem to be competitive in forming vesicle clusters in nodules, that is, does not fix nitrogen in nodules, and that two such strains were identified in two attempts out of eight, suggests that not all strains that can colonize *D. glomerata* nodules can form vesicles in infected cells. In other words, *D. glomerata* is not very efficient in discriminating against symbiotically inefficient *Frankia* strains.

### Average Nucleotide Identity Comparisons Show That Cppng1_Ca_nod Represents a Novel Species

**Fig. 2.**
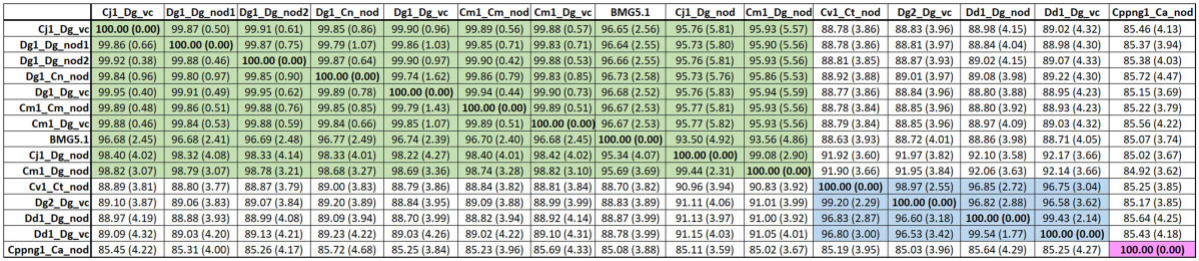
—Mean ANI values between (meta-)genomes of *Frankia* cluster-2. Comparisons between Eurasian/Mediterranean (meta-)genomes are labeled in green, comparisons between North American metagenomes are labeled in blue. The Southern hemisphere metagenome (Cppng1_Ca_nod) is labeled in pink. SDs are given in brackets.

In order to find out whether the new (meta-)genomes represented novel species of *Frankia* cluster-2, Average Nucleotide Identity (ANI) comparisons were performed for the 13 genomes of the cluster. The mean ANI values shown in figure 2 show that if the newly calculated ANI threshold range of 98.65% as equivalent of 70% DNA–DNA hybridization were applied (Kim et al. 2014), the inoculum from Alaska (Dd1_Dg_vc and Dd1_Dg_nod) would represent a new species. However, based on the usually applied ANI threshold range of 95–96% for species demarcation set by Goris et al. (2007) and Richter and Rosselló-Móra (2009), all North American inocula available thus far represent members of *Candidatus* Frankia californiensis (Normand et al. 2017). For the Eurasian strains, strong sequence conservation is found between *Candidatus* Frankia datiscae Dg1 (Persson et al. 2011) represented by Dg1_Dg_v, Dg1_Dg_nod1, Dg1_Dg_nod2, and Dg1_Cn_nod, as well as Cv1_Dg_vc, Cm1_Dg_vc, and Cm1_Cm_nod. As already seen in the core genome phylogeny (fig. 1), *Frankia coriariae* BMG5.1 (Gtari et al. 2015; Nouioui, Ghodhbane-Gtari, Rohde, et al. 2017) shows higher similarity with *Candidatus* Frankia datiscae than the two metagenomes Cm1_Dg_nod and Cv1_Dg_nod.

The genome sequenced from the Southern hemisphere inoculum, Cppng1_Ca_nod, has <86% ANI with all other *Frankia* cluster-2 (meta-)genomes available. Thus, based on ANI values, the Southern hemisphere strain Cppng1_Ca_nod represents a novel species of cluster-2 *Frankia*. Therefore, we propose this strain as type strain of a new species, *Candidatus* Frankia meridionalis (me.riː.di.oːˈnaː.lis. N.L. masc./fem. adj. meridionalis, southern). The species nodulates and fixes nitrogen in the root nodules of *Coriaria* species growing in Papua New Guinea and New Zealand and is genetically different from all other Cluster-2 *Frankia* strains sequenced so far. It also could nodulate the Northern hemisphere species *C.terminalis* (supplementary fig. S4, Supplementary Material online).

Altogether, while the differences between strains of the same inoculum observed in this study were still within the species level, there was significant strain diversity within an inoculum. Nevertheless, the composition of the _vc versions, which represent ∼10 g of nodules each, implies that only (a) particular strain(s) can form vesicle clusters in a particular host plant species. Furthermore, the striking sequence conservation among the _vc version of Eurasian (meta-)genomes, that is, between Cm1_Dg_vc from France, Cj1_Dg_vc from Japan and Dg1_Dg_vc from Pakistan cannot, in contrast with the genetic conservation of *Casuarina*-infective strains (fig. 1), be ascribed to geographic isolation. This phenomenon might instead be ascribed to the low saprotrophic potential of cluster-2 *Frankia*. While there is evidence that cluster-2 strains can occasionally occur in the soil in the absence of their host plant species, this seems to be rare (Battenberg et al. 2017).

### *Frankia* Cluster-2 Genome Instability: Transposase Abundance in the Different Species

**Fig. 3.**
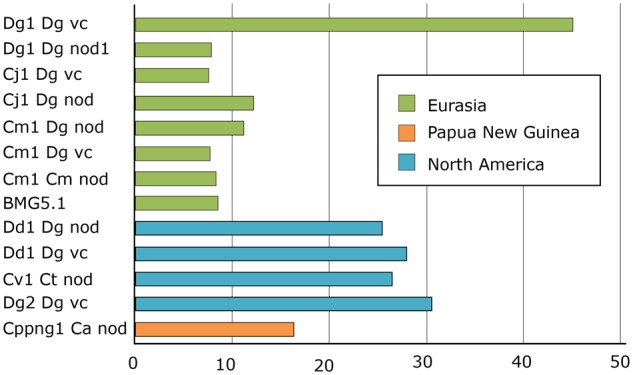
—Transposase abundance. Numbers of transposase ORFs per Mbp (meta-)genome in the cluster-2 *Frankia* (meta-)genomes are given. The genome of *Candidatus* Frankia Datiscae Dg1 sequenced from DNA isolated from vesicle clusters (Dg1_Dg_vc) contains at least four times more transposases in its genome than all other Eurasian genomes; this discrepancy is explained by the fact that this genome is the only one in the group which is not in draft stage, but fully assembled. Among the draft genomes, the Eurasian strains contain significantly less transposase ORFs than the North American strains (Wilcoxon’s *P* = 0.006).

Although genome stability is vital for the survival of any organism, a certain flexibility is required in order to adapt to a changing environment. Bacterial genomes achieve this flexibility using mobile DNA elements, horizontal gene transfer, and genome rearrangement (Juhas et al. 2009). In particular, insertion sequence (IS) elements confer genomic plasticity (Mugnier et al. 2009; Raeside et al. 2014). Previous analyses had shown that the genome of the cluster-2 strain Dg1 contained more full size IS elements—that is, IS elements capable of transposition—than genomes of representatives of the other *Frankia* clusters (Persson et al. 2015). Therefore, transposase abundance was examined in all cluster-2 (meta-)genomes. The results are depicted in figure 3.

The genome of *Candidatus* Frankia Datiscae Dg1 sequenced from DNA isolated from vesicle clusters (Dg1_Dg_vc) contains at least four times more transposases in its genome than all other Eurasian genomes; this discrepancy is explained by the fact that this genome is the only one in the group which is not in draft stage, but fully assembled. This result underlines the fact that transposase abundance can be compared between draft genomes or between fully assembled genomes, but not between a mixture of both. Since genome assembly usually condenses all copies of an insertion element into a single contig, fully assembled genomes will contain more repetitive elements than draft genomes. The results for the draft (meta-)genomes obtained in this study, including Dg2 (Nguyen et al. 2016), clearly show that the North American strains, that is, representatives of the species *Candidatus* Frankia californiensis (Normand et al. 2017), contain significantly more IS elements than the Eurasian strains, that is, the representatives of the species *Candidatus* Frankia datiscae (Persson et al. 2015) and *Frankia coriariae* BMG5.1 (Nouioui, Ghodhbane-Gtari, Rohde, et al. 2017). The number of IS elements in the Southern hemisphere metagenome Cppng1_Ca_nod seems to be closer to those of the Eurasian than of the North-American (meta-)genomes, but since so far the Southern hemisphere species is represented by a single strain, conclusions might be premature.

### All Sequenced Eurasian *Frankia* Cluster-2 (Meta-)Genomes except for BMG5.1 Contain the Canonical *Nod* Genes *nodABC*, All (Meta-)Genomes from North America Also Contain *nodH*

The (meta-)genomes of *Candidatus* Frankia datiscae Dg1 and *Candidatus* Frankia californiensis Dg2 contained the canonical *nod* genes *nodABC* that are responsible for the synthesis of the common backbone of rhizobial symbiotic signal factors, LCOs (Persson et al. 2015; Nguyen et al. 2016). Dg2 additionally contained the sulfotransferase gene *nodH*. However, no *nod* genes were found in the genome of the only cluster-2 strain cultured thus far, BMG5.1 (Gtari et al. 2015). As listed in table 1, all but one of the (meta-)genomes sequenced in this study contain the canonical *nod* genes *nodABC*. All North American metagenomes also contained *nodH*. The exception is the Southern hemisphere metagenome, Cppng1_Ca_nod, which contained *nodC*, a truncated copy of *nodB2*, and no *nodA*.

### The *nodA’B1A Operon* (nod1 Region) Is Part of a Transposable Unit That Also Contains a *betAdegT* Operon

**Fig. 4.**
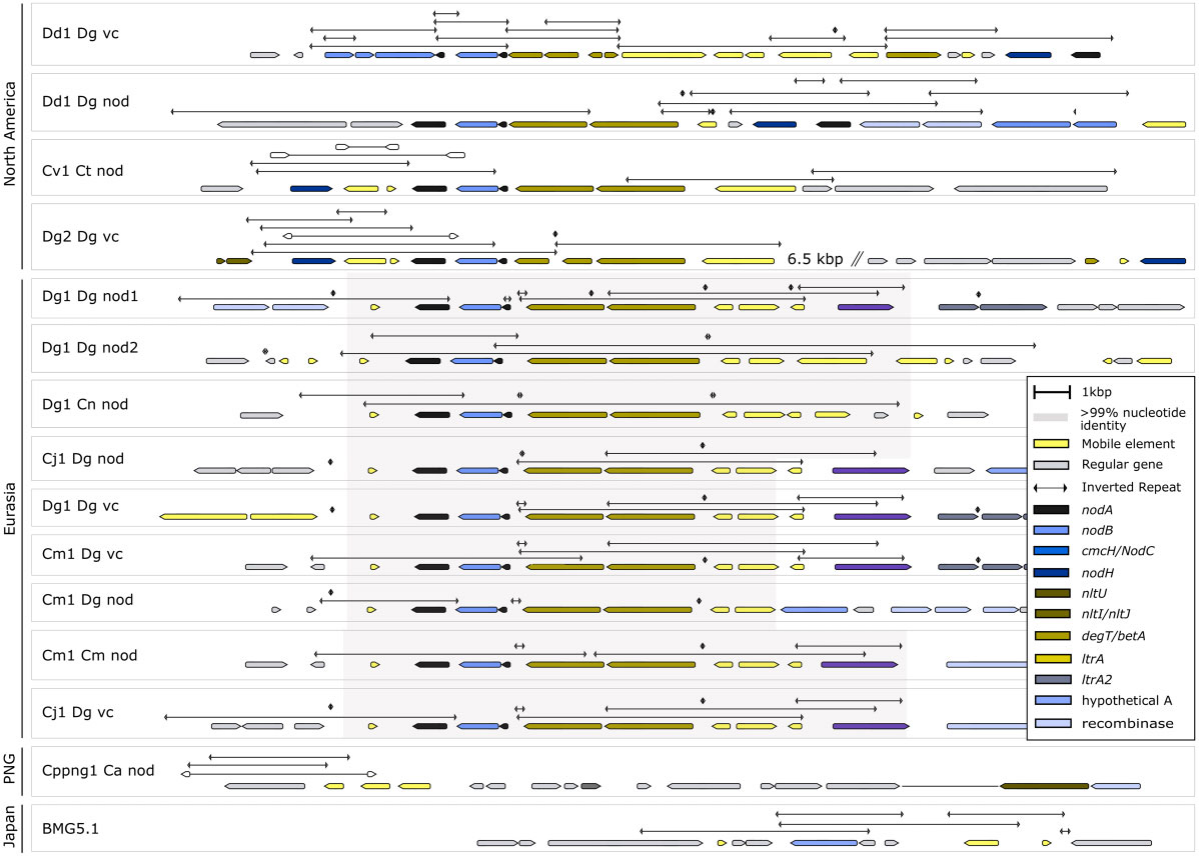
—*NodA’B1A* operons (nod1 region) in different (meta-)genomes. Illustration of the nod1 region of the *Frankia* cluster-2 (meta-)genomes available. This region contains the *nodAB1A'degTbetA* genes, enclosed by transposases. In all Eurasian strains, this region shows >99% sequence conservation (shaded in pink). The exceptions are two small insertions involving repeated CTAG tetramers, and a transposition: in Cm1_Dg_nod, a downstream transposase is replaced by a gene of unknown function, referred to as encoding hypothetical protein A. This gene is also found in Cj1_Dg_nod and BMG5.1, always adjacent to the gene *padR*. In Cppng_Ca_nod and BMG5.1, the *nodAB1A'degTbetA* region is missing; instead, suggested regions for its excision are shown. The sequence conservation as well as the surrounding transposases and inverted repeats suggests that the *nodAB1A'degTbetA* region is mobile. Inverted repeats of interest are indicated as double-pointed arrows. A version of this figure with gene names above the ORFs is available in the supplementary fig. S5, Supplementary Material online.

In Dg1, the canonical *nod* genes were present as two operons in different locations on the chromosome, *nodA’B1A* (nod1 region) and *nodB2CnltIJ* (nod2 region; also contained homologs of the nodulation-related ABC transporter genes *nodI* and *nodJ*). Synteny analysis showed that in all Eurasian *Frankia* strains with the exception of BMG5.1 and BMG5.30, the *nodA’B1A* operon is part of a consensus region (shaded in pink in fig. 4). This region includes transposases on both sites of the *betAdegT nodA’B1A* operons. Nucleotide identity in this region is 100% for Eurasian (meta-)genomes with three exceptions (fig. 4). In the North American metagenomes, the nod1 region synteny has been affected by more transposition events leading, for example, to the transposition of *nodA* from the *nodA’B1A* operon in Dd1_Dg_vc while a truncated copy of *nodA* was retained in the operon, or simply to the duplication of *nodA* in Dd1_Dg_nod.

### The *nodB2CnltIJ* Operon (nod2 Region) Is Enclosed by Inverted Repeats and Might Represent a Transposable Unit

**Fig. 5.**
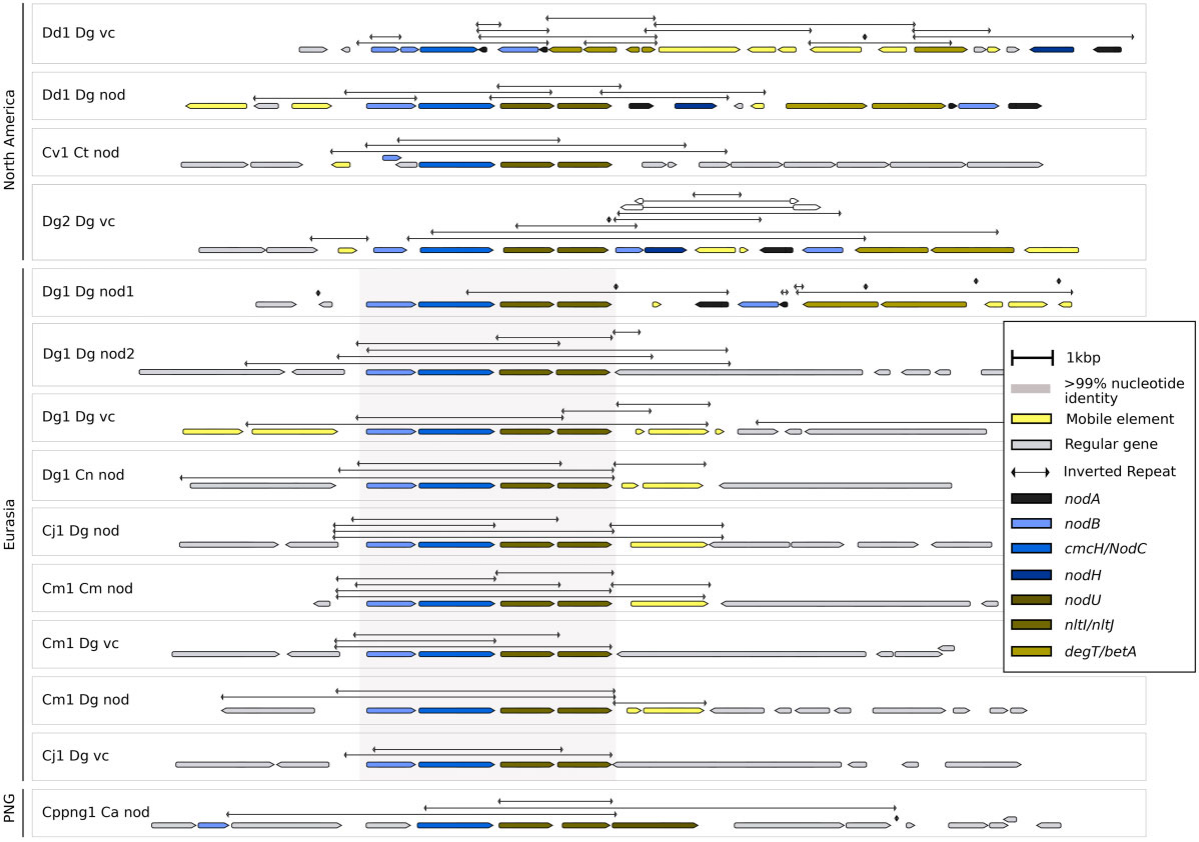
—*NodB2CnltIJ* operon (nod2 region) in different (meta-)genomes. Illustration of the nod2 region of all cluster-2 *Frankia* (meta-)genomes available (BMG5.1 is not included here since it does not contain the nod2 region). This part of the genome contains the *nodB2CnltIJ* genes (shaded in pink). Like the nod1 region, the nod2 region is identical in the Eurasian (meta-)genomes, except for 18 bp between the *nod* and *nlt* genes involving repeated CTAG tetramers. In Dg2_Dg_vc, the region is located between two transposases and adjacent to an area rich in inverted repeats which may have been involved in the creation of the *nodB1H-transposase-nodA’B* region. Inverted repeats of interest are indicated as double-pointed arrows. A version of this figure with gene names above the ORFs is available in the supplementary fig. S5, Supplementary Material online.

The nod2 region sequences are also highly conserved in the Eurasian (meta-)genomes (shaded in pink in fig. 5), except for 18 bp between the *nodB2C* and *nltIJ* operons where the sequences differ due to insertions or deletions of the inverted repeat CTAGCTAGCTAG. Again, the sequences of the nod2 regions of the North American strains are less conserved and appear to have acquired multiple point mutations. In Dg2_Dg_vc (Nguyen et al. 2016) as well as in Dd1_Dg_vc and Dd1_Dg_nod, the nod1 and nod2 regions are linked. In these three metagenomes, there is at least one IS elements between the two linked regions.

The nod2 region is enclosed by inverted repeats (CTAGCTAGCTAG; fig. 5). No obvious mobile genetic element can be found upstream of the *nodB2CnltIJ* region of the Eurasian (meta-)genomes, but several strains have transposases immediately downstream of the region.

### The Cppng1_Ca_nod Metagenome Contains an Operon with the *nodB2’CnltIJ* Genes and a Homolog of the Carbamoyl Transferase Encoding Gene *nodU*

The metagenome originating from *C. papuana* nodules collected in Papua New Guinea did not contain an *nodA’B1A* operon (nod1 region), but it did contain an operon containing a truncated copy *nodB2* and complete copies of *nodC* and *nltIJ* (nod2 region; fig. 5). Interestingly, two ORFs were present between *nodB2* and *nodC*, CPPNG1CANOD_5034 and CPPNG1CANOD_5033. The proteins encoded by these two ORFs had no homologs with >5e^−26^ with proteins encoded by any other of the cluster-2 (meta-)genomes known thus far, or with proteins encoded by any other publicly available *Frankia* genome. Nevertheless, they represent members of actinobacterial protein families with close homologs present in several other actinobacterial genomes (closest homolog with 5e^−178^ for CPPNG1CANOD_5034 and with 1e^−66^ for CPPNG1CANOD_5033). The closest homolog of the 259 amino acid transmembrane protein Cppng1_Ca_nod_5034 was PBC69544 from *Streptomyces* sp. CF124 (JGI) which was isolated from the *Populus* root microbiome. The closest homolog of the 636 amino acid cytosolic protein Cppng1_Ca_nod_5033 was WP_020524966 from *Catelliglobosispora koreensis* (JGI) which was isolated from a gold mine cave (Ara et al. 2008). *NodC* is followed by *nltIJ* which is followed by a gene predicted to encode a carbamoyl transferase.

**Fig. 6.**
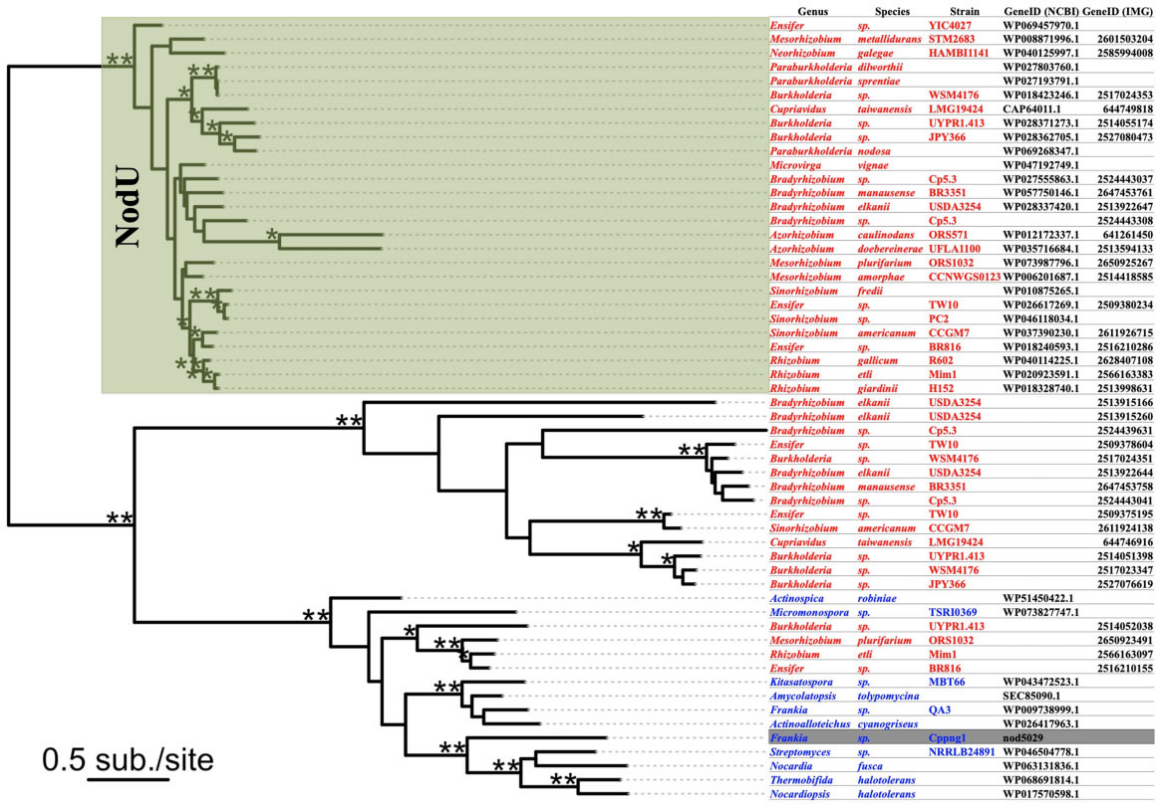
—Carbamoyl transferase phylogeny. Rhizobial strain designations are given in red, actinobacterial strain designations in blue. The sequence from Cppng1_Ca_nod is highlighted in gray. The phylogram shows that rhizobial NodU proteins form a distinct clade, while the non-NodU carbamoyltransferases group in two clades, one of which also includes all actinobacterial sequences examined. *denotes nodes with >90 bootstrap support; **denotes nodes with 100 bootstrap support.

This carbamoyl transferase has the highest homology (starting with *E* = 0.0) with carbamoyl transferases from the secondary metabolism of actinobacteria, hydroxymethyl cephem carbamoyltransferases (*cmcH*; Aharonowitz et al. 1992). Among its homologs are also the rhizobial NodU proteins, carbamoyltransferases involved in the chemical decoration of Nod factors. Therefore, the phylogeny of actinobacterial hydroxymethyl cephem carbamoyltransferases and alpha-proteobacterial carbamoyl transferases was examined (fig. 6).

The phylogenetic tree (fig. 6) consisted of three major clades each with high bootstrap support. One of the three clades included all known rhizobial NodU proteins with one rhizobial protein that had not explicitly been described as NodU. All actinobacterial sequences including the *Candidatus* Frankia meridionalis Cppng1 carbamoyl transferase belonged to another clade, which was the only one of the three clades that included both rhizobial and actinobacterial sequences. Thus, the Cppng1 gene does not have the same origin as rhizobial *nodU* genes. However, based on its linkage to *nodB2’C*, we are calling the Cppng1 gene “*nodU-*like” *(nltU)* in this article.

### Expression of *Nod* Genes in Symbiosis

**Fig. 7.**
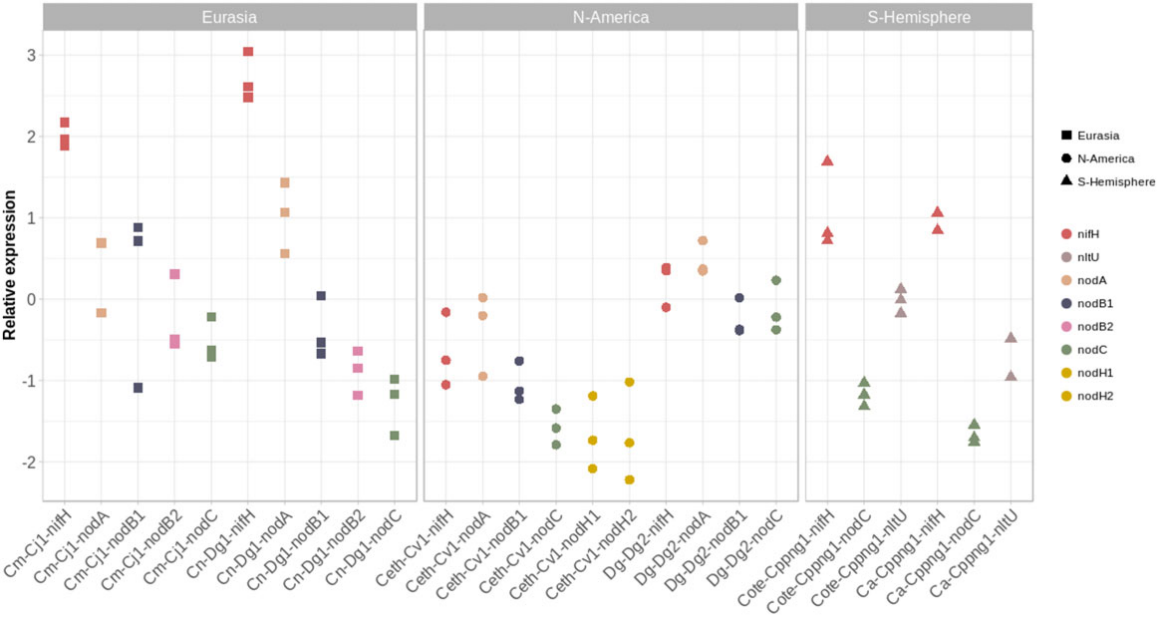
—*Nod* gene expression in nodules. Relative expression levels were determined based on *IF-3* as reference gene (Alloisio et al. 2010) and presented at log10 scale. Expression levels of the nitrogenase structural gene *nifH* were included in the analysis as a further point of comparison. Results are listed based on initials of host plant-name of inoculum-name of gene. Host plants were *Coriaria myrtifolia* (Cm), *C. nepalensis* (Cn), *C. terminalis* (Cote), *C. arborea* (Ca), *Ceanothus thyrsiflorus* (Ceth), and *Datisca glomerata* (Dg). The figure was prepared using R (R Core Team 2019).

Previous studies had shown that *nodB1A* and *nodB2C* are expressed in nodules of *D. glomerata* while *nodH1* and *nodH2* are not (Persson et al. 2015; Nguyen et al. 2016). Therefore, the expression of *nod* genes was examined in nodules induced on *Cea. thyrsiflorus* by Cv1, nodules of *C. myrtifolia* induced by Cj1, nodules of *C. nepalensis* induced by Dg1, and in nodules of *C. arborea* and *C. terminalis* induced by Cppng1, as well as in nodules of *D. glomerata* induced by Dg2. The results are depicted in figure 7. *NodB1A* and *nodB2C* were expressed in all host plants from the Northern Hemisphere, while *nodC* and *nltU* were expressed in nodules of *C. arborea* and *C. terminalis*. *NodH* was expressed in nodules of *Cea. thyrsiflorus*. Thus, *nodH* expression seemed to be host-specific in that the gene was not expressed in nodules of *D. glomerata* (Datiscaceae, Cucurbitales) induced by Dg2 which contains *nodH*, but was expressed in nodules of *Cea. thyrsiflorus* (Rhamnaceae, Rosales) induced by Cv1.

### What Is the Function of *Frankia* Cluster-2 *Nod* Genes?

Two facts point toward a role of *Frankia nod* genes in determining host specificity: first, *nodH* shows host-specific expression, and second, all *Frankia* cluster-2 metagenomes from North America, that is, from the area of host plants from the Rosales order, contain *nodH*. With the exception of the Southern Hemisphere metagenome Cppng1_C1_nod, the *nod* gene regions display various signs of earlier transposition events (figs. 3 and 4). Nevertheless, the *nod* regions of Eurasian strains show strong sequence conservation, while the *nod* regions of North American strains show less conservation; the latter fact could be linked to the increased transposase abundance in North American compared with Eurasian (meta-)genomes (fig. 2). The facts that 1) the two *nod* operons *nodA’B1A* and *nodB2CnltIJ* are part of transposable units and that 2) both operons are linked in two of the North American metagenomes (Dg2_Dg_vc and Dd1_Dg_vc) and in one of the metagenomes based on inoculum Dg1 from Pakistan (Dg1_Dg_nod1) suggest that in the common progenitor of *Frankia* cluster-2 all *nod* genes were linked.

The fact that BMG5.1 and BMG5.30 do not contain the canonical *nod* genes (Gtari et al. 2015; Gueddou et al. 2019) shows that *nod* gene-free strains can be part of cluster-2 assemblages. This is not surprising given the fact that the *nod* genes are present on transposable units in all Northern Hemisphere (meta-)genomes sequenced so far; indeed, it seems likely that all cluster-2 strain assemblages would contain *nod* gene-free member(s). BMG5.1 and BMG5.30 can nodulate *C.myrtifolia* (Gtari et al. 2015; Gueddou et al. 2019). Thus, the lack of *nod* genes is no obstacle to nodulation of *Coriaria* sp.

However, the fact that all Northern hemisphere (meta-) genomes described in this study, nine of which were propagated in *D. glomerata* nodules, contain the canonical *nod* genes seems to imply that the *nod* gene-free strains do not compete very well with *nod* gene-containing strains when it comes to infection of *D. glomerata*. In our hands, BMG5.1 could not nodulate *D. glomerata* while it could nodulate *C. myrtifolia*; this contradicts the results of Gtari et al. (2015), who reported nodulation of both species by BMG5.1. The discrepancy might be explained by the fact that Gtari et al. (2015) studies were performed in a Mediterranean climate which *D. glomerata* is adapted to.

A role of cluster-2 *Frankia nod* genes in the nodulation of *Datisca* sp. and of the North American host plants, but not of *Coriaria* sp., would also be consistent with the fact that the Southern hemisphere metagenome Cppng1_Ca_nod, which does not contain functional copies of *nodA* and *nodB*, could nodulate *C. arborea* and *C. terminalis* but was unable to nodulate *Datisca* sp. However, the fact that Cppng1_Ca_nod *nodC* and *nltU* were expressed in nodules of *C. arborea* could be interpreted to mean that the function of *Frankia* cluster-2 *nod* genes is not, or not only, the synthesis of LCOs, but, for example, the modification of N-acetylglucosamine-containing phospholipids that occur in actinobacteria (Sun et al. 2015). In any case, the fact that also in all *nod* gene containing cluster-2 strains including Cppng1_Ca_nod, the nod2 region includes *nltIJ*, the operon encoding part of an ABC transport system, suggests that the enzymes encoded by the *nod* genes are involved in producing molecules for export. Altogether, further research is needed to clarify the function of *Frankia* cluster-2 *nod* genes, but the data available thus far support a function in the nodulation of all cluster-2 host plants except for Coriariaceae.

### Evolution of *Frankia* Cluster-2 Symbioses

All plants able to enter a root nodule symbiosis with nitrogen-fixing soil bacteria go back to a common ancestor (Soltis et al. 1995), and there are two hypotheses on the evolution of root nodule symbioses. In the first one, ∼100 Ma the common ancestor of the Rosales, Cucurbitales, Fagales, and Fabales acquired a predisposition based on which a root nodule symbiosis could evolve, and the evolution of such symbioses then took place in several lineages for *Frankia*, and in several lineages for rhizobia as microsymbionts (Soltis et al. 1995; Doyle 2016). The second hypothesis assumes that the common ancestor of the four orders evolved a symbiosis with nitrogen-fixing soil bacteria, which in turn was lost in the majority of lineages; this hypothesis is supported by phylogenomic analyses (Griesmann et al. 2018; van Velzen et al. 2018) and therefore will be the basis of this discussion. Since the distribution of the Fagales has been studied in detail, leading to the conclusion that the order evolved in Gondwana (Cook and Crisp 2005), it can be concluded that the common ancestor of plants forming a nitrogen-fixing root nodule symbiosis (Fagales, Fabales, Rosales, and Cucurbitales) evolved in the Gondwana supercontinent.

With regard to host specificity it is interesting that the inoculum from the Southern hemisphere, Cppng1 could not only nodulate *C. arborea* but also the Northern Hemisphere species *C. terminalis*, while it failed to nodulate *D. glomerata*. This is to our knowledge the first report on the nodulation of a Northern Hemisphere *Frankia* cluster-2 host plant by an inoculum from the Southern Hemisphere. Previous attempts with *Purshia* sp. and *Ceanothus* sp. as host plants were unsuccessful (Silvester 1977; Benson and Silvester 1993), and nodulation of *C. arborea* by the Northern Hemisphere inoculum Dg1 failed as well (data not shown). So while the fact that *Frankia* cluster-2 inocula represent strain assemblages is complicating the interpretation of cross-infection studies, it is worth noting that *Coriaria* is the only host plant genus distributed in both hemispheres, and so far, the only successful cross-hemisphere inoculation involved a *Coriaria* species.

The fact that most inocula used in this study were propagated in nodules of *D. glomerata* might have biased the outcome, both with regard to the members of the strain assemblages that were sequenced, and with regard to the results of cross-inoculation studies. The distribution of *Coriaria* sp. and *D.cannabina* overlaps in northern India/Pakistan/Nepal and it temporarily overlapped in France since *D. cannabina* used to be cultivated there (Stenhouse 1856). That is, in these areas, selection could have favored cluster-2 inocula able to nodulate *D. cannabina* and *Coriaria* spp. Their distribution does/did not, however, currently or in the recorded past, overlap in Japan; yet, the Cj1 inoculum could nodulate *D. glomerata*.

As outlined by Nguyen et al. (2016), cluster-2 *Frankia* strains probably reached North America from Asia during the Eocene/Oligocene (55 − 25 Ma) with *Datisca* sp. over the Beringian land bridge. In the Southern hemisphere, currently cluster-2 *Frankia* strains can only nodulate *Coriaria* species. In Eurasia, currently they can be hosted by *Coriaria* species and also by *D.cannabina*. When *Datisca* sp. arrived in North America—allopatric speciation between *D. cannabina* and *D. glomerata* has been dated to 25 Ma (Zhang et al. 2006)—*Frankia* cluster-2 strains from Eurasia encountered the microsymbionts of the North American host plants, Dryadoideae and *Ceanothus* sp., all from the Rosales order. The phylogenetic position of the metagenomes from the North American inocula (fig. 1), one of which was isolated from *Cea. thyrsiflorus*, suggests that the Eurasian lineage of *Frankia* cluster-2 outcompeted the endogenous North American cluster-2 strains. Again, the results presented here may be biased based on the use of *D. glomerata* to propagate inocula. Nevertheless, the fact that the Cv1 metagenome isolated from nodules *of Cea. thyrsiflorus* maps in the same lineage as the three North American metagenomes that were propagated in *D. glomerata*, supports the hypothesis that the original Rosales lineage(s) of cluster-2 *Frankia* strains was/were outcompeted. Data on transposase abundance indicate that this process was associated with increased transposition rates, that is, increased genomic instability in the *Datisca* lineage of cluster-2 *Frankia* strains.

## Conclusions

The analysis of 12 new (meta-)genomes of *Frankia* cluster-2 strains based on two previously used and five novel inocula showed that cluster-2 inocula represent groups of strains. This might explain their wide host range. A comparison of (meta-)genomes based on DNA isolated from whole nodules versus DNA isolated from symbiotic bacterial structures (vesicle clusters) showed that strains that are strongly underrepresented in vesicle clusters may still be able to colonize nodules.

The analysis of transposases in the available (meta-)genomes showed that North American strains contain more transposases than Eurasian strains. An analysis of the evolution and distribution of host plants indicates that bursts of transposition may have coincided with the expansion of the host range while outcompeting the endogenous cluster-2 strains.

All novel (meta-)genomes contained the canonical *nod* genes *nodABC*; the North American ones also contained the sulfotransferase gene *nodH*. An analysis of the synteny of the nod regions indicated that they were located on mobile genetic elements. This would lead to the expectation that groups of cluster-2 strains would also contain members that had lost the canonical *nod* genes, like the only cultured cluster-2 strain *F. coriariae* BMG5.1 (Gtari et al. 2015).

So far, the presence of *nodABC* in a cluster-2 *Frankia* (meta-)genome is correlated with the ability to nodulate *D. glomerata*, and *nodH* was found only in genomes originating in North America. *NodABC* expression was found in all nodules examined, while *nodH* was expressed only in *Cea. thyrsiflorus* (Rhamnaceae, Rosales), not in *D. glomerata* (Datiscaceae, Cucurbitales). However, more data are needed to confirm the hypothesis that cluster-2 *Frankia nod* genes are involved in host specificity.

The first metagenome of an inoculum from the Southern hemisphere was obtained, Cppng1_Ca_nod. The strain represents a novel cluster-2 *Frankia* species and does not contain the canonical *nod* genes *nodAB*, but still contains *nodC*. It could nodulate a northern hemisphere *Coriaria* species, *C. terminalis*. 

## Supplementary Material

Supplementary data are available at *Genome Biology and Evolution* online.

## Supplementary Material

evz153_Supplementary_Data

## Notes

**Data deposition:** This project has been deposited at EMBL/GenBank/DDBJ under the accession PRJEB19438 - PRJEB19449.
