# Supplementary material for: Frankia-Enriched Metagenomes from the Earliest Diverging Symbiotic Frankia Cluster: They Come in Teams
Source: Genome Biol Evol. 2019 Jul 19;11(8):2273–91. doi: 10.1093/gbe/evz153 (PMC6735867; doi:10.1093/gbe/evz153)
Supplement: evz153_Supplementary_Data [file evz153_supplementary_data.zip › Suppl legends.docx]

**Supplementary Material online**

**Supplementary Table S1. Inocula used in this study.**

**Supplementary Table S2. Genomes and accession numbers of the corresponding BioProjects in GenBank.**

**Supplementary Table S3. BUSCO analysis of *Frankia*-enriched metagenomes.**

**Supplementary Table S4. Primers sequences used to analyse *nifH and nodA, nodB1, nodB2, nodC, nodH1, nodH2,* and *nodU* expression.**

**Supplementary Fig. S1. Localisation of DNA in *Ceanothus thyrsiflorus* nodule.** (**a,b**) Nodule lobe overwiev. The meristem (m) at the tip of the nodule lobe is responsible for the devleopmental gradient of the infected cells (ic) in the cortex, where they are interspersed with uninfected cells (uc). The vascular system is surrounded by a multi-layered pericycle (p). (**c,d**) Infected cells in the cortex with hhe vesicles (arrows) stained with DAPI (red channel), indicating that they contain DNA, just like the hyphae in the infected cell in the middle that contains a large nucleus (n).  **(a, c)** A single optical section of a differential interference contrast microscopy image is combined with a red channel showind DNA stained with DAPI. Bars: **(a, b)** 100 µm, **(c, d)** 20 µm.

**Supplementary Fig. S2. Comparison of Cj1_Dg_nod and Cm1_Dg_nod with the respective _vc versions. (a, b)** BUSCO analysis with statistics. The plot was created by means of BUSCO plot (v2.0; Simã*o* et al. 2015). The different categories of Busco and their proportion in the data sets are shown. **(c, d)** BlastN comparison of the binned contigs of Cj1 Dg nod and Cm1 Dg nod *vs.* the corresponding draft genomes of their respective _vc versions. The sequence similarity is on the x-axis, while the y-axis indicates the amount of hits.

**Supplementary Fig. S3. Attempt to quantify strains in Dg1_Dg_nod2 (a) and Dg1_Cn_nod (b): *Frankia* SNP distribution in correlation to genome position.** Large plots show the local SNP distribution for the *Frankia* draft genomes of Dg1_Cn_nod and Dg1_Dg_nod2. The percentage of SNPs is given on the y-axis of each plot and the positions are given on the x-axis. Genomic regions characterized by a SNP frequency of <10% represent less abundant SNPs or sequencing errors. The amount of SNPs makes clear that the samples contains more than one strain each, but no conclusion can be drawn with regard to the number of strains.

**Supplementary Fig. S4. Nodule induced by the inoculum from Papua New Guinea, Cppng1, on *Coriaria terminalis.***

**Supplementary Figure S5. *NodA’B1A* operons (nod1 region) and *nodB2CnltIJ* operon (nod2 region) in different *Frankia*-enriched metagenomes.** Enlargeable version of Figure 4 and Figure 5.

**Supplementary File S1.** Scripts and data used.
