# Supplementary material for: Frankia-Enriched Metagenomes from the Earliest Diverging Symbiotic Frankia Cluster: They Come in Teams
Source: Genome Biol Evol. 2019 Jul 19;11(8):2273–91. doi: 10.1093/gbe/evz153 (PMC6735867; doi:10.1093/gbe/evz153)

Supplementary Fig. S1. Localisation of DNA in *Ceanothus thyrsiflorus* nodule. (**a,b**) Nodule lobe overwiev. The meristem (m) at the tip of the nodule lobe is responsible for the devleopmental gradient of the infected cells (ic) in the cortex, where they are interspersed with uninfected cells (uc). The vascular system is surrounded by a multi-layered pericycle (p). (**c,d**) Infected cells in the cortex with hhe vesicles (arrows) stained with DAPI (red channel), indicating that they contain DNA, just like the hyphae in the infected cell in the middle that contains a large nucleus (n). **(a, c)** A single optical section of a differential interference contrast microscopy image is combined with a red channel showind DNA stained with DAPI. Bars: **(a, b)** 100 µm, **(c, d)** 20 µm.

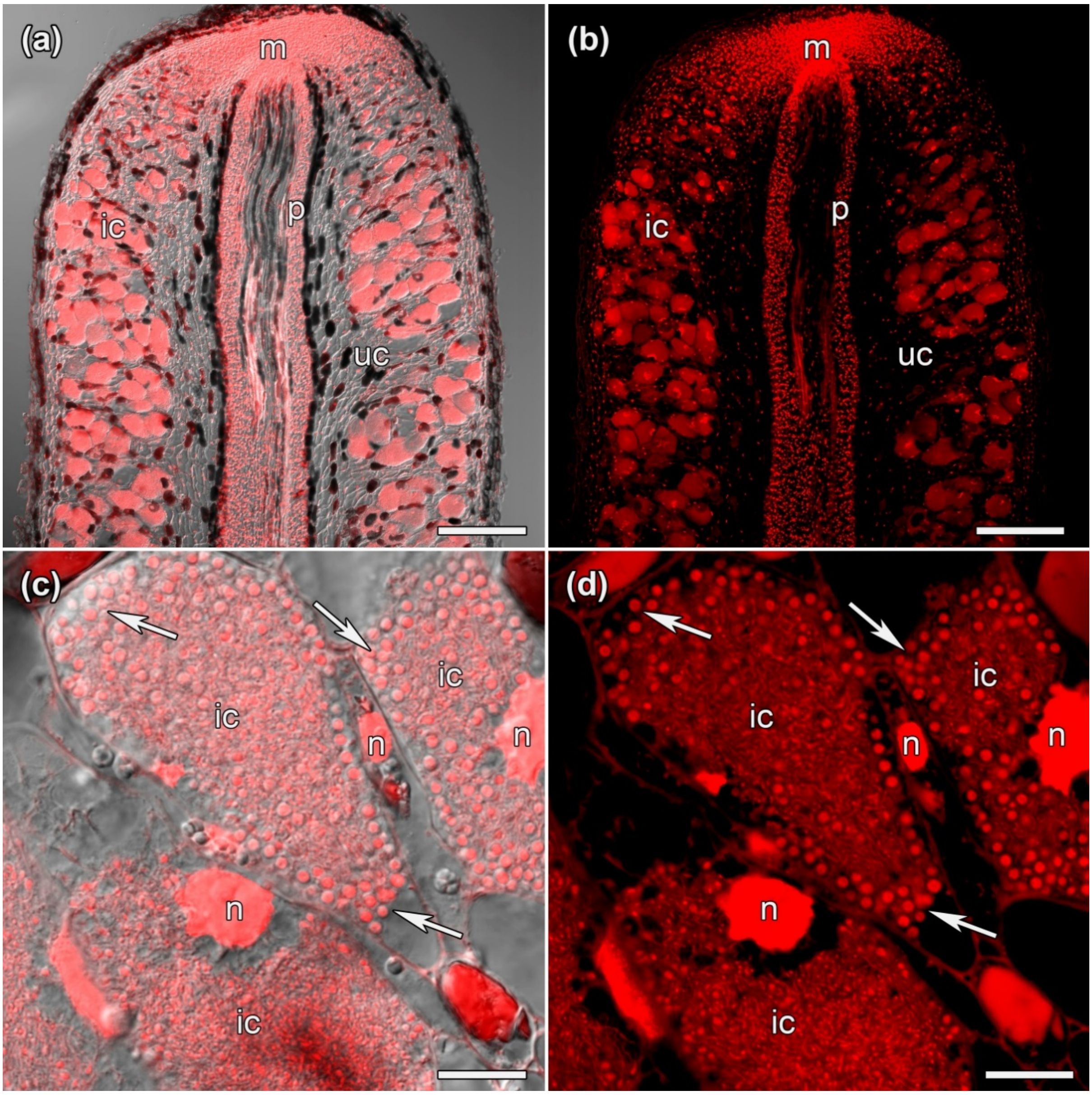

Supplement: evz153_Supplementary_Data [file evz153_supplementary_data.zip › supplementary Fig S1--OLD.pdf]
