# Supplementary material for: Frankia-Enriched Metagenomes from the Earliest Diverging Symbiotic Frankia Cluster: They Come in Teams
Source: Genome Biol Evol. 2019 Jul 19;11(8):2273–91. doi: 10.1093/gbe/evz153 (PMC6735867; doi:10.1093/gbe/evz153)

Supplementary Fig. S2. Comparison of Cj1_Dg_nod and Cm1_Dg_nod with the respective _vc versions. (a, b) BUSCO analysis with statistics. The plot was created by means of BUSCO plot (v2.0; Simão et al. 2015). The different categories of Busco and their proportion in the data sets are shown. **(c, d)** BlastN comparison of the binned contigs of Cj1 Dg nod and Cm1 Dg nod *vs.* the corresponding draft genomes of their respective _vc versions. The sequence similarity is on the x-axis, while the y-axis indicates the amount of hits.

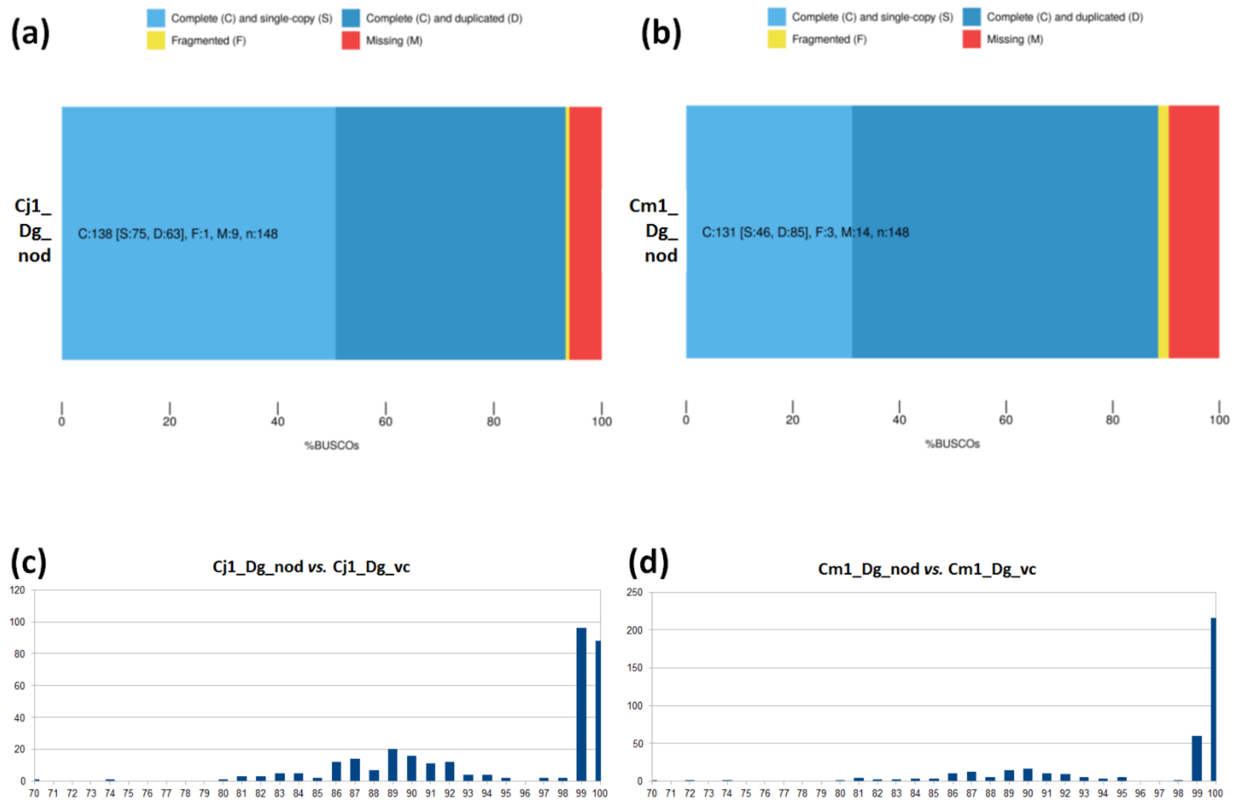

Supplement: evz153_Supplementary_Data [file evz153_supplementary_data.zip › Supplementary Fig S2.pdf]
