# Supplementary material for: Frankia-Enriched Metagenomes from the Earliest Diverging Symbiotic Frankia Cluster: They Come in Teams
Source: Genome Biol Evol. 2019 Jul 19;11(8):2273–91. doi: 10.1093/gbe/evz153 (PMC6735867; doi:10.1093/gbe/evz153)

Supplementary Fig. S3. Attempt to quantify strains in Dg1_Dg_nod2 (a) and Dg1_Cn_nod (b): *Frankia* SNP distribution in correlation to genome position. Large plots show the local SNP distribution for the *Frankia* draft genomes of Dg1_Cn_nod and Dg1_Dg_nod2. The percentage of SNPs is given on the y-axis of each plot and the positions are given on the x-axis. Genomic regions characterized by a SNP frequency of <10% represent less abundant SNPs or sequencing errors. The amount of SNPs makes clear that the samples contains more than one strain each, but no conclusion can be drawn with regard to the number of strains.

(a)

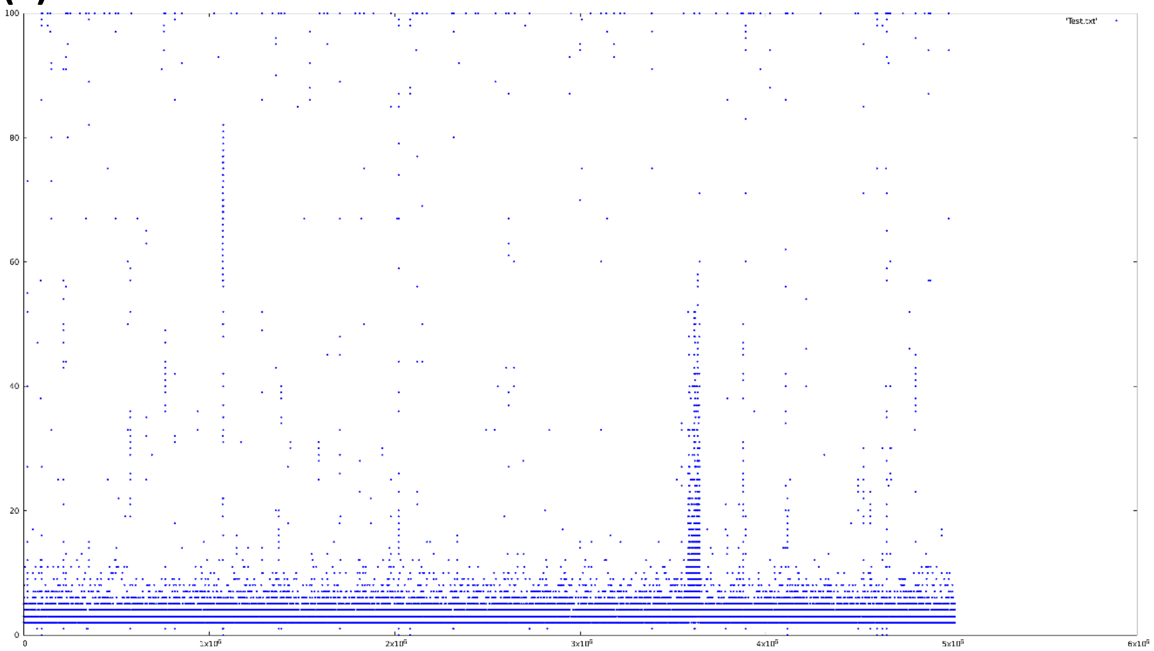

(b)

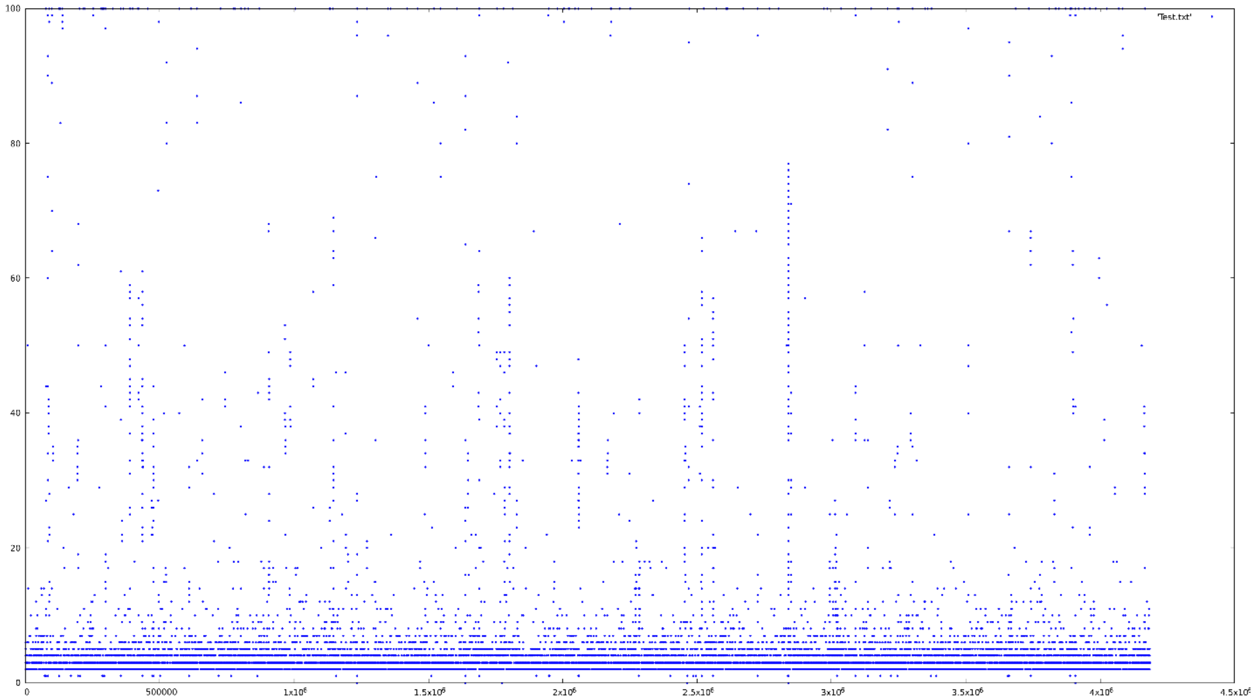

Supplement: evz153_Supplementary_Data [file evz153_supplementary_data.zip › Supplementary Fig S3.pdf]
