# Supplementary material for: Frankia-Enriched Metagenomes from the Earliest Diverging Symbiotic Frankia Cluster: They Come in Teams
Source: Genome Biol Evol. 2019 Jul 19;11(8):2273–91. doi: 10.1093/gbe/evz153 (PMC6735867; doi:10.1093/gbe/evz153)

Supplementary Fig. S4. Nodule induced by the inoculum from Papua New Guinea, Cppng1, on *Coriaria terminalis.*

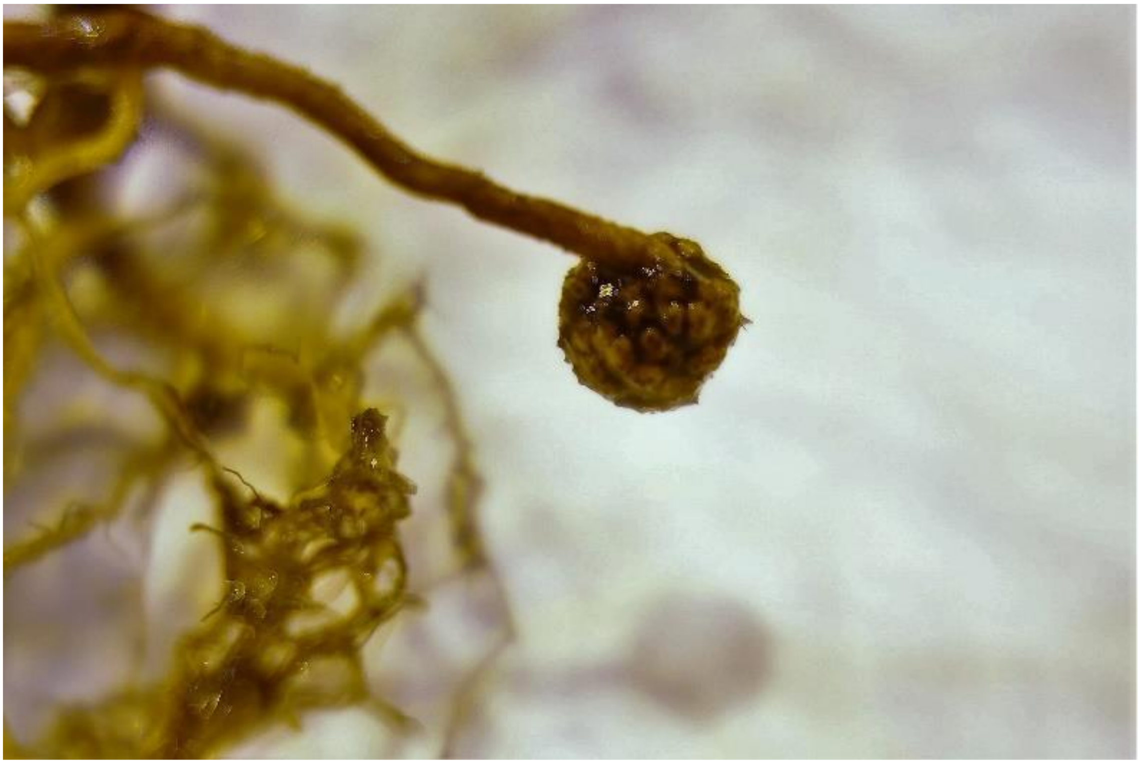

Supplement: evz153_Supplementary_Data [file evz153_supplementary_data.zip › Supplementary Fig S4new.pdf]
