# Supplementary material for: Frankia-Enriched Metagenomes from the Earliest Diverging Symbiotic Frankia Cluster: They Come in Teams
Source: Genome Biol Evol. 2019 Jul 19;11(8):2273–91. doi: 10.1093/gbe/evz153 (PMC6735867; doi:10.1093/gbe/evz153)
Supplement: evz153_Supplementary_Data [file evz153_supplementary_data.zip › Supplementary File 1.pdf]

Supplementary File 1. Scripts and data used.

```
################################################################
#
#   Autothink.py
#   Written by theoden.vigil-stenman@su.se
#
#  - Identifies transposase sequences by repeated blastx searches aginst a database of
transposase aa sequences
#   - Joins neighboring hits whose aa sequences complement each other
#   - Output in genbank file
#
#   The program has no user interface, variables need to be adjusted in the script:
#   results_directory,genome_directory,blast_program_directory,isblastdbfile need to be set
correctly
#
#   Needs the file classholder.py (in path or same directory as autothink.py)
#
#################################################################

import os
from Bio.Blast.Applications import NcbiblastxCommandline
from Bio import SeqIO
from os.path import join
import sqlite3
from Bio.Blast import NCBIXML
from Bio.Seq import Seq
from Bio.Alphabet import IUPAC
from Bio.SeqRecord import SeqRecord
from Bio.SeqFeature import SeqFeature, FeatureLocation
import pickle
from classholder import IsPair

# Removes the old features to replace with "joined" features
def removeFeature_for_curation(featureslist,toberemoved):
    newfeatures=[]
    for oldfeat in featureslist:
        if oldfeat.location.start==toberemoved.location.start and
oldfeat.location.end==toberemoved.location.end and
oldfeat.qualifiers["is_name"]==toberemoved.qualifiers["is_name"]:
            continue
        else:
            newfeatures.append(oldfeat)
    return newfeatures

# Checks dictionary to see if position is already treated
def none_in_range_for_curation(filename, featurelocation):
    if filename in none_in_range_dic.keys():
        for entry in none_in_range_dic[filename]:
            if entry[0]==min(int(featurelocation.start),int(featurelocation.end)) and
entry[1]==max(int(featurelocation.start),int(featurelocation.end)):
                return "none_in_range"
    return "none_in_dic"

# Finds the closest feature of the right type
def findClosest_for_curation(myfeature, gbfile,maxdistance):
    dist_to_features_fh=open(gbfile,"r")
    features_to_check_distance_to=list(SeqIO.parse(dist_to_features_fh,"genbank"))[0]
    dist_to_features_fh.close()
    closefeaturesObs=[]
    for feature in features_to_check_distance_to.features:
        if feature.type in islist:
            checkstart= int(feature.location.start)
            checkend =int(feature.location.end)
            if checkstart==myfeature.location.start and checkend==myfeature.location.end:
                continue
            checkstart_start=abs(checkstart-int(myfeature.location.start))
            checkstart_end=abs(checkstart-int(myfeature.location.end))
            checkend_start=abs(checkend-int(myfeature.location.start))
```

```
            checkend_end=abs(checkend-int(myfeature.location.end))
            mindistance=min(checkstart_start,checkstart_end,checkend_start,checkend_end)
            shortest_distance="wrong_in_choose_shortest_distance"
            if checkstart_start==mindistance:
                if checkstart-int(myfeature.location.start)>0:
                    shortest_distance="start_newstart"
                else:
                    shortest_distance="newstart_start"
            elif checkstart_end==mindistance:
                if checkstart-int(myfeature.location.end)>0:
                    shortest_distance="end_newstart"
                else:
                    shortest_distance="newstart_end"
            elif checkend_start==mindistance:
                if checkend-int(myfeature.location.start)>0:
                    shortest_distance="start_newend"
                else:
                    shortest_distance="newend_start"
            elif checkend_end==mindistance:
                if checkend-int(myfeature.location.end)>0:
                    shortest_distance="newend_end"
                else:
                    shortest_distance="end_newend"
            if mindistance<maxdistance:
                closefeaturesObs.append(IsPair(myfeature,feature,mindistance,shortest_distance))
    sortedclosefeaturesObs=sorted(closefeaturesObs,key=lambda x: x.getMinDistance(),
reverse=False)
    return sortedclosefeaturesObs

# Puts transposases with no neighbors in a file, so searches don't have made over and over
def append_to_nir_dic_for_curation(full_file_name,feature_location):
    if full_file_name in none_in_range_dic.keys():

none_in_range_dic[full_file_name].append([min(int(feature_location.start),int(feature_location.en
d)),max(int(feature_location.start),int(feature_location.end))])
    else:

none_in_range_dic[full_file_name]=[[min(int(feature_location.start),int(feature_location.end)),ma
x(int(feature_location.start),int(feature_location.end))]]

def parse_gb_file_for_curation(full_file_name,orgname,read_pickle_dic,aminoacid_distance):
    gb_fh=open(full_file_name,"r")
    parsed=list(SeqIO.parse(gb_fh,"genbank"))
    gb_fh.close()
    if len(parsed)!=1:
        raw_input("number of records in file is not one")
    else:
        for record in parsed:
            featurelist=[]
            s=Seq(str(record.seq),IUPAC.IUPACUnambiguousDNA())
            newrec=SeqRecord(s)
            newrec.id=record.id
            newrec.description=record.description
            for feature in record.features:
                if feature.type in islist:
                    closest=findClosest_for_curation(feature,full_file_name,maxdistance)
                    if closest==[]:
                        if read_pickle_dic==False:
                            append_to_nir_dic_for_curation(orgname,feature.location)
                        continue
                    elif closest[0].getOpposingStrands():
                        continue
                    aaboutdistance=closest[0].getClosestDistance()[1]
                    aadistance=abs(int(aaboutdistance[0][0])-int(aaboutdistance[1][0]))
                    if aadistance<aminoacid_distance:

newfeatures=removeFeature_for_curation(record.features,closest[0].getNewCloseFeature())

newfeatures2=removeFeature_for_curation(newfeatures,closest[0].getOrigCloseFeature())

                        drawstart=closest[0].getStartOfBoth()
```

```
                        drawend=closest[0].getEndOfBoth()
                        newfeature = SeqFeature(FeatureLocation(drawstart,drawend),
strand=closest[0].getStrand(),type="joinedhit")

                        newfeature.id="joined"
                        newfeature.qualifiers["is_name"]=[closest[0].getName()]
                        newfeature.qualifiers["sbjct_start"]=[closest[0].get_joined_aa_start()]
                        newfeature.qualifiers["sbjct_end"]=[closest[0].get_joined_aa_end()]
                        newfeature.qualifiers["orflength"]=[closest[0].get_aa_orflength()]
                        newfeature.qualifiers["ntlength"]=[closest[0].getShortestNt()]
                        newrec.features.append(newfeature)

                        newfeatures2.append(newfeature)
                        newrec.features=newfeatures2
                        return newrec

        return "no_joins"

# Get part of nucleotide sequence
def getseq(start,end,seq):
    return seq[start-1:end]

def doblast(isseq,offset):
    # Write query to temporary file
    writequery=open(tempfile,"w")
    writequery.write(">test\n"+str(isseq))
    writequery.close()
    blastline=blast_program_directory+"blastx -db "+isblastdbfile+" -out "+tempfileout+" -query
"+tempfile+" -query_gencode 11 -num_threads 4 -outfmt 5 -evalue "+ str(mineval_first_search)
    os.system(blastline)
    try:
        blastout_handle=open(tempfileout,"r")
        blast_records = list(NCBIXML.parse(blastout_handle))
    except:
        print "blast parse trouble in function getbestblast"
        raw_input()
        return None
    #collect all hsp hits in one file
    hsps=[]
    for record in blast_records:
        for alg in record.alignments:
            for hsp in alg.hsps:
                # Collect hit info in dictionary
                dic={}
                dic["hit_def"]=str(alg.hit_def)
                dic["sbjct_start"]=hsp.sbjct_start
                dic["match"]=hsp.match
                dic["identities"]=hsp.identities
                dic["positives"]=hsp.positives
                dic["sbjct_end"]=hsp.sbjct_end
                dic["expected"]=str(hsp.expect)
                dic["frame"]=hsp.frame
                dic["bits"]=hsp.bits
                dic["query"]=str(hsp.query)
                dic["mod_query_end"]=hsp.query_end+offset-1
                dic["mod_query_start"]=hsp.query_start+offset-1
                dic["query_end"]=hsp.query_end
                dic["query_start"]=hsp.query_start
                dic["sbjct"]=str(hsp.sbjct)
                dic["score"]=hsp.score
                dic["align_length"]=hsp.align_length
                dic["query_length"]=record.query_length
                dic["queried_seq"]=isseq

dic["hit_seq"]=getseq(int(min(int(hsp.query_start),int(hsp.query_end))),int(max(int(hsp.query_sta
rt),int(hsp.query_end))),isseq)
                hsps.append(dic)
    mysortedhsps=sorted(hsps,key=lambda x: x["score"], reverse=True)
    if len(mysortedhsps)>0:
        return mysortedhsps[0]
    else:
```

```
        return None

def find_starts_ends(querylist):
    lookingfor="start"
    results=[]
    fcounter=1
    currentstart=-1
    for i in querylist:
        if lookingfor=="start":
            if i==1:
                currentstart=fcounter
                lookingfor="end"
                fcounter+=1
                continue
        if lookingfor=="end":
            if i==0:
                results.append([currentstart,fcounter-1])
                lookingfor="start"
        fcounter+=1
    if lookingfor=="end":
        results.append([currentstart,len(querylist)])
    return results

def parse_xml_file(sample,cur):
    replies=[]
    xmlfh=open(sample,"r")
    try:
        blast_records = list(NCBIXML.parse(xmlfh))
    except:
        raw_input("no file")
    if len(list(blast_records))<1:
        raw_input("no records")
    parseitrecordcounter=0
    for rec in blast_records:
        parseitrecordcounter+=1
        hit_def="NONE"
        mycsvstring=""
        gbhitslist=[]
        if (len(rec.alignments))<1:
            print "parseit: no algs for "+samplename
            continue
        hit_name=((str(rec.query)).split(" "))[0]
        cur.execute('''SELECT seq FROM genomes WHERE readid=?''', (hit_name,))
        ans=cur.fetchall()
        queryseq=ans[0][0].encode('ascii','ignore')

        # make genbank record
        s=Seq(str(queryseq),IUPAC.IUPACUnambiguousDNA())
        print "parseit: got seq, len"+str(len(queryseq))
        newrec=SeqRecord(s)
        newrec.id= str(rec.query).split(" ",1)[0]
        if len(str(newrec.id))>15:
            newrec.id= str(rec.query)[0:16]
        if len(str(rec.query).split(" "))>1:
            newrec.description= str(rec.query).split(" ",1)[1]
        else:
            newrec.description= rec.query
        allfeatures=[]
        querylist=[0]*rec.query_length
        algs=rec.alignments
        # Get coverage of hits
        for alg in algs:
            for hsp in alg.hsps:
                if hsp.expect<mineval_first_search:
                    querystart=min(hsp.query_start,hsp.query_end)
                    queryend=max(hsp.query_start,hsp.query_end)
                    querylist[querystart-1:queryend]=(1+queryend-querystart)*[1]
                    feature = SeqFeature(FeatureLocation(hsp.query_start,hsp.query_end),
strand=int(hsp.frame[1]),type="firsthit")
                    feature = SeqFeature(FeatureLocation(hsp.query_start,hsp.query_end),
strand=0,type="firsthit")
```

```
                    feature.id=alg.hit_id
                    feature.id="test"
                    feature.qualifiers["hit"]=alg.hit_def
                    allfeatures.append(feature)
        starts_and_ends=find_starts_ends(querylist)

        for st_end in starts_and_ends:
            feature = SeqFeature(FeatureLocation(st_end[0],st_end[1]), strand=1,type="footprint")
            allfeatures.append(feature)

        # Blast coverages
        for res in starts_and_ends:
            footprintseq=queryseq[res[0]-1:res[1]]
            firstsearch=[footprintseq,1,len(footprintseq)]
            remainsearches=[firstsearch]
            while len(remainsearches)>0:
                currentsearch=remainsearches.pop()
                currentseq=currentsearch[0]
                oldstart=currentsearch[1]
                oldend=currentsearch[2]
                if len(currentseq)>4:
                    doblast_results=doblast(currentseq,res[0])
                else:
                    doblast_results=None
                if (doblast_results==None) or
(float(doblast_results["expected"])>mineval_id_searches):
                    pass
                else:

blast_hitstart=min(int(doblast_results["query_start"]),int(doblast_results["query_end"]))

blast_hitend=max(int(doblast_results["query_start"]),int(doblast_results["query_end"]))
                    recorded_blast_hitstart=blast_hitstart+oldstart-1
                    recorded_blast_hitend=blast_hitend+oldstart-1

listToAppend=[recorded_blast_hitstart+res[0],recorded_blast_hitend+res[0],doblast_results["hit_de
f"],doblast_results,res[0]]
                    gbhitslist.append(listToAppend)
                    if not blast_hitstart==1:
                        leftstart=oldstart
                        leftend=oldstart+blast_hitstart-2

leftremains_list=[getseq(leftstart,leftend,footprintseq),leftstart,leftend]
                        remainsearches.append(leftremains_list)
                    if not blast_hitend==len(currentseq):
                        rightstart=oldstart+blast_hitend
                        rightend=oldend

rightremains_list=[getseq(rightstart,rightend,footprintseq),rightstart,rightend]
                        remainsearches.append(rightremains_list)
        sortedhits=sorted(gbhitslist,key=lambda x: x[3]["score"], reverse=True)

        for hit in sortedhits:
            if hit[3]["frame"][0]>=1:
                hitstrand=1
            if hit[3]["frame"][0]<0:
                hitstrand=-1
            splitname=hit[2].split("__")
            orflength=splitname[6].replace("orflength:","")
            subjstart=int(hit[3]["sbjct_start"])
            subjend=int(hit[3]["sbjct_end"])
            aahitlen=1+max(subjstart,subjend)-min(subjstart,subjend)
            perc_of_orf=round(aahitlen/float(orflength),3)
            minorf=min(subjstart,subjend)
            maxorf=max(subjstart,subjend)
            orftype="IS"
            if detailed_orfnames:
                if perc_of_orf>=complete_cutoff:
                    orftype="completeIS"+"_"+str(complete_cutoff)
                elif perc_of_orf>=0.8:
                    orftype="wholeIS"+"_"+str(whole_cutoff)
```

```
                elif minorf<=float(orflength)*isstart_start_cutoff and
maxorf<=float(orflength)*isstart_end_cutoff:
                    orftype="ISstart"
                elif minorf>=float(orflength)*isend_start_cutoff:
                    orftype="ISend"
                elif minorf>=float(orflength)*ismiddle_start_cutoff and
maxorf<=float(orflength)*ismiddle_end_cutoff:
                    orftype="ISmiddle"

            feature = SeqFeature(FeatureLocation(int(hit[0]),int(hit[1])),
strand=hitstrand,type=orftype)
            feature.id=hit[3]["hit_def"]
            family=splitname[1].replace("family:","")
            group=splitname[2].replace("group:","")
            origin=splitname[3].replace("origin:","")
            accession=splitname[4].replace("accession:","")
            ntlength=splitname[5].replace("ntlength:","")
            isnumorfs=splitname[7].replace("isnumorfs","")
            feature.qualifiers["is_name"]=splitname[0]
            feature.qualifiers["family"]=family
            feature.qualifiers["group"]=group
            feature.qualifiers["origin"]=origin
            feature.qualifiers["ntlength"]=ntlength
            feature.qualifiers["orflength"]=orflength
            feature.qualifiers["numorfs"]=isnumorfs
            feature.qualifiers["sbjct_start"]=str(hit[3]["sbjct_start"])
            feature.qualifiers["sbjct_end"]=str(hit[3]["sbjct_end"])
            feature.qualifiers["expected"]=str(hit[3]["expected"])
            feature.qualifiers["score"]=str(hit[3]["score"])
            feature.qualifiers["query_length"]=str(hit[3]["query_length"])
            #feature.qualifiers["match"]=str(hit[3]["match"])
            feature.qualifiers["query_start"]=str(hit[3]["query_start"])
            feature.qualifiers["identities"]=str(hit[3]["identities"])
            feature.qualifiers["align_length"]=str(hit[3]["align_length"])
            feature.qualifiers["positives"]=str(hit[3]["positives"])
            #feature.qualifiers["query"]=str(hit[3]["query"])
            #feature.qualifiers["queried_seq"]=str(hit[3]["queried_seq"])
            feature.qualifiers["query_end"]=str(hit[3]["query_end"])
            feature.qualifiers["frame"]=str(hit[3]["frame"])
            feature.qualifiers["bits"]=str(hit[3]["bits"])
            #feature.qualifiers["sbjct"]=str(hit[3]["sbjct"])
            feature.qualifiers["mod_query_start"]=str(hit[3]["mod_query_start"])
            feature.qualifiers["mod_query_end"]=str(hit[3]["mod_query_end"])
            feature.qualifiers["perc_of_orf"]=str(perc_of_orf)
            allfeatures.append(feature)
        newrec.features=allfeatures
        replies.append([hit_name,newrec,mycsvstring,samplename])
    return replies

def maketablemakestring(tablename):
    makestring="CREATE TABLE "+tablename+"("
    makestring+="readid"+" "+"TEXT"+","
    makestring+="seq"+" "+"TEXT"+","
    makestring+="seqlen"+" "+"INT"+","
    makestring=makestring[:-1]
    makestring+=")"
    return makestring

##############################
#   VARIABLES
##############################

results_directory="/Users/security/Documents/Katharina/21febtest2/"

# Directory with genbank files
genome_directory="/Users/security/Documents/Katharina/newgenomes_mod/"
# Directory for produced blast xml files
blast_results_directory=results_directory+"xmlfiles/"
# Location of blast program
blast_program_directory="/Users/security/blast/ncbi-blast-2.2.30+/bin/"
# Directory for genbank results files
```

```
parse_results_dir=results_directory+"parsed_files/"
# Location of database to be made
genome_db_destination=results_directory+"genomedb.db"
# Name of table in database
sqlite_tablename="genomes"
# Locations of temporary files
tempfile=results_directory+"temp_blastfile.fa"
tempfileout=results_directory+"temp_idblastresult.xml"
# Location of blast database
isblastdbfile="/Users/security/is_aa_30_nov2016.fa"
# Directory for curated files
curated_directory=results_directory+"curated/"

pickledic=results_directory+"autocurate_pickled_dick.pydic"


# First blastx search cutoff
maximum_evalue=0.0001
# Number of hits to record
max_targets=10000
# cutoff for footprint
mineval_first_search=1e-4
# cutoff for id
mineval_id_searches=1e-4

# Annotate hits as start, end, middle?
detailed_orfnames=True
# cutoff for "complete" annotation
complete_cutoff=0.95
# cutoff for "whole" annotation
whole_cutoff=0.8
# To be considered to be a "start", hit start position is below X * protein aa length
isstart_start_cutoff=0.3
# To be considered to be an "start", hit end position is below X * protein aa length
isstart_end_cutoff=0.5
# To be considered to be an "end", hit start position is above X * protein aa length
isend_start_cutoff=0.7
# To be considered to be an "middle", hit start and end positions are between X * protein aa
length and Y* protein aa length
ismiddle_start_cutoff=0.3
ismiddle_end_cutoff=0.7

# Names of files to be treated
sample_list=["cci3.gb","cj1dgvc.gb","dd1dgvc.gb","ean1pec.gb","cm1cmnod.gb","dg1cnnod.gb","eui1c.
gb","cm1dgnod.gb","dg1dgnod.gb","acn14a.gb","cm1dgvc.gb","dg1dgvc.gb","mchersina44151.gb","crpngc
anod.gb","dg2dgvc.gb","bmg51.gb","cv1ctnod.gb","dg2dgvc_nodregion.gb","cj1dgnod.gb","dd1dgnod.gb"
,"newdg1dgnod.gb"]

sample_list=["newdg1dgnod.gb","crpngcanod.gb"]
#sample_list=["dg1dgnod2.gb"]
#sample_list=["dg1cnnod2.gb"]

# List of feature types to be treated
islist=["IS","ISstart","ISend","ISmiddle","wholeIS_0.8","completeIS_0.95","joinedhit"]

# Max distance between hits to be considered for joining
maxdistance=1000
none_in_range_dic={}



##################################
#   START OF PROGRAM EXECUTION
##################################


if not os.path.exists(results_directory):
    os.makedirs(results_directory)
if not os.path.exists(blast_results_directory):
    os.makedirs(blast_results_directory)
if not os.path.exists(parse_results_dir):
```

```
    os.makedirs(parse_results_dir)
if not os.path.exists(curated_directory):
    os.makedirs(curated_directory)

# Make connections for genome database
makegenome_con = sqlite3.connect(genome_db_destination)
makegenome_cur = makegenome_con.cursor()

# Go through each sample in sample_list
for samplename in sample_list:
    sample=genome_directory+samplename
    if ".gbk" in sample or ".gb" in sample:
        fh=open(sample,"r")
        parsed_genbank=list(SeqIO.parse(fh,"genbank"))
        if len(list(parsed_genbank))==1:
            # blastx search against database

destination=blast_results_directory+samplename.replace(".gbk","").replace(".gb","")+".xml"
            SeqIO.write(parsed_genbank[0],open(genome_directory+"temp.fa","w"),"fasta")
            blastx_cline = NcbiblastxCommandline(query=genome_directory+"temp.fa",
db=isblastdbfile, evalue=maximum_evalue, outfmt=5,
out=destination,max_target_seqs=max_targets,num_threads=4,query_gencode=11)
            os.system(blast_program_directory+str(blastx_cline))
            # put genome in database
            genome_dropstring="DROP TABLE IF EXISTS "+sqlite_tablename
            genome_makestring=maketablemakestring(sqlite_tablename)
            with makegenome_con:
                    makegenome_cur.execute(genome_dropstring)
                    makegenome_cur.execute(genome_makestring)
            record=parsed_genbank[0]
            readid=record.id
            seq= str(record.seq)
            seqlen=len(seq)
            insertline="INSERT INTO "+sqlite_tablename+" VALUES('"
            insertline+=readid+"','"
            insertline+=seq+"',"
            insertline+=str(seqlen)+","
            insertline= insertline[:-1]
            insertline= insertline+")"
            with makegenome_con:
                makegenome_cur.execute(insertline)
            parseresults=parse_xml_file(destination,makegenome_cur)
        else:
            raw_input("zero or more than 1 record in genbank file")
        for parseresult in parseresults:
            hit_name=parseresult[0]
            gbrec=parseresult[1]
            csvline=parseresult[2]
            mysamplename=parseresult[3]
            gbparseddir=parse_results_dir+samplename+"_is.gbk"
            genbank_output=open(gbparseddir,"w")
            SeqIO.write([gbrec],genbank_output,"genbank")
            genbank_output.close()
        # Make dictionary for curation
        counter=0
        while parseresults!="no_joins":
            counter+=1
            if counter>1:
                break
            parseresults=parse_gb_file_for_curation(gbparseddir,samplename,False,50)
            pickle.dump(none_in_range_dic, open(pickledic,"wb"))
        # Perform the actual curation
        counter=0
        infile=join(gbparseddir)
        while parseresults!="no_joins":
            counter+=1
            parseresults=parse_gb_file_for_curation(infile,samplename,True,50)
            if not parseresults=="no_joins":
                outfile=join(curated_directory,samplename)
                outfh=open(outfile,"w")
                SeqIO.write([parseresults],outfh,"genbank")
```

```
                outfh.close()
                infile=outfile

#################################################################
#
#   classholder.py
#   Written by theoden.vigil-stenman@su.se
#
#   This is a collection of utility classes for the script autothink.py
#
##################################################################

import os
from Bio.SeqFeature import SeqFeature, FeatureLocation
from Bio.Seq import Seq
from Bio.Alphabet import IUPAC
from Bio import SeqIO
from Bio.SeqRecord import SeqRecord

class InvertFinder:

    def __init__(self):
        self.testvar="here we are, testing"
        self.tempfile="/Users/security/invertfindertemp.fa"
        self.outfile="/Users/security/invertfindertemp_outfile.fa"
        self.outseq="/Users/security/invertfindertemp_outseq.fa"

    def getIRs(self,seq,maxrepeat):
        gap_penalty=12
        min_score_threshold=50
        match_score=3
        mismatch_score=-4
        irfile=self.makefasta(seq)
        irfinderstring="einverted -sequence "+irfile+" -gap "+str(gap_penalty)+" -threshold
"+str(min_score_threshold)+" -match "+str(match_score)+" -mismatch "+str(mismatch_score)+ " -
outfile "+self.outfile+ " -outseq "+self.outseq+ " -maxrepeat "+str(maxrepeat)
        #print irfinderstring
        os.system(irfinderstring)

    def makefasta(self,seq):
        s=Seq(str(seq),IUPAC.IUPACUnambiguousDNA())
        newrec=SeqRecord(s)
        newrec.id="invertfindertemp"
        newrec.description=""
        newrec.name=""
        tempfilefh=open(self.tempfile,"w")
        SeqIO.write([newrec],self.tempfile,"fasta")
        tempfilefh.close()
        return self.tempfile

class IsPair:
    def __init__(self,orig_feature,new_feature,mindistance,shortest_distance):
        self.orig_feature=orig_feature
        self.new_feature=new_feature
        self.mindistance=mindistance
        self.shortest_distance=shortest_distance

self.newOrfLenght=(int(orig_feature.qualifiers["orflength"][0])+int(new_feature.qualifiers["orfle
ngth"][0]))/2
        if shortest_distance=="end_newstart":
            if orig_feature.strand==1:

aareturns=[[self.orig_feature.qualifiers["sbjct_end"][0],"old_end_aa"],[self.new_feature.qualifie
rs["sbjct_start"][0],"new_start_aa"]]
                self.meeting_end=self.orig_feature.qualifiers["sbjct_end"][0]
                self.meeting_start=self.new_feature.qualifiers["sbjct_start"][0]
                self.joined_aa_start=self.orig_feature.qualifiers["sbjct_start"][0]
                self.joined_aa_end=self.new_feature.qualifiers["sbjct_end"][0]
            elif orig_feature.strand==-1:
                self.meeting_end=self.new_feature.qualifiers["sbjct_end"][0]
                self.meeting_start=self.orig_feature.qualifiers["sbjct_start"][0]
```

```
                self.joined_aa_start=self.new_feature.qualifiers["sbjct_start"][0]
                self.joined_aa_end=self.orig_feature.qualifiers["sbjct_end"][0]
        elif shortest_distance=="newend_start":
            if orig_feature.strand==1:
                self.meeting_end=self.new_feature.qualifiers["sbjct_end"][0]
                self.meeting_start=self.orig_feature.qualifiers["sbjct_start"][0]
                self.joined_aa_start=self.new_feature.qualifiers["sbjct_start"][0]
                self.joined_aa_end=self.orig_feature.qualifiers["sbjct_end"][0]
            elif orig_feature.strand==-1:
                self.meeting_end=self.orig_feature.qualifiers["sbjct_end"][0]
                self.meeting_start=self.new_feature.qualifiers["sbjct_start"][0]
                self.joined_aa_start=self.orig_feature.qualifiers["sbjct_start"][0]
                self.joined_aa_end=self.new_feature.qualifiers["sbjct_end"][0]
        else:
            raw_input("third type"+shortest_distance)

    def getShortestType(self):
        return self.shortest_distance
    def get_aa_orflength(self):
        return self.newOrfLenght
    def get_joined_aa_start(self):
        return self.joined_aa_start
    def get_joined_aa_end(self):
        return self.joined_aa_end
    def getStrand(self):
        return self.orig_feature.strand
    def getMinDistance(self):
        return self.mindistance
    def getNewCloseFeature(self):
        return self.new_feature
    def getOrigCloseFeature(self):
        return self.orig_feature
    def getOpposingStrands(self):
        if self.orig_feature.strand!=self.new_feature.strand:
            return True
        else:
            return False
    def getClosestDistance(self):
        aareturns=[[],[]]
        aareturns="NOOOOO"
        if self.getShortestType()=="newstart_start":

returns=[int(self.new_feature.location.start),int(orig_feature.location.start),"newstart_start"]
        elif self.getShortestType()=="start_newstart":

returns=[int(self.orig_feature.location.start),int(new_feature.location.start),"start_newstart"]
        elif self.getShortestType()=="newstart_end":

returns=[int(self.new_feature.location.start),int(orig_feature.location.end),"newstart_end"]
        elif self.getShortestType()=="end_newstart":
            if self.orig_feature.strand==1:

aareturns=[[self.orig_feature.qualifiers["sbjct_end"][0],"old_end_aa"],[self.new_feature.qualifie
rs["sbjct_start"][0],"new_start_aa"]]
            if self.orig_feature.strand==-1:
                aareturns=[[self.orig_feature.qualifiers["sbjct_start"][0],"old start
aa"],[self.new_feature.qualifiers["sbjct_end"][0],"new_end_aa"]]

returns=[int(self.orig_feature.location.end),int(self.new_feature.location.end),"end_newstart"]
        elif self.getShortestType()=="start_newend":

returns=[int(self.orig_feature.location.start),int(self.new_feature.location.end),"start_newend"]
        elif self.getShortestType()=="newend_start":
            if self.orig_feature.strand==1:

aareturns=[[self.new_feature.qualifiers["sbjct_end"][0],"new_end_aa"],[self.orig_feature.qualifie
rs["sbjct_start"][0],"old_start_aa"]]
            if self.orig_feature.strand==-1:

aareturns=[[self.new_feature.qualifiers["sbjct_start"][0],"new_end_aa"],[self.orig_feature.qualif
iers["sbjct_end"][0],"old_start_aa"]]
```

```
returns=[int(self.new_feature.location.end),int(self.orig_feature.location.start),"newend_start"]
        elif self.getShortestType()=="end_newend":

returns=[int(self.orig_feature.location.end),int(self.new_feature.location.end),"end_newend"]
        elif self.getShortestType()=="newend_end":

returns=[int(self.new_feature.location.end),int(self.orig_feature.location.end),"newend_end"]
        else:
            returns=[0,0,"bokren"]
            raw_input("broken")
        return [returns,aareturns]
    def getOrigName(self):
        return str(self.orig_feature.qualifiers["is_name"][0])
    def getNewName(self):
        return str(self.new_feature.qualifiers["is_name"][0])
    def getName(self):
        nameslist=[str(self.getOrigName()).replace(" ",""),str(self.getNewName()).replace("
",""))]
        nameslist.sort()
        return nameslist[0]+"|"+nameslist[1]
    def getStartOfBoth(self):
        location_of_start=int(self.orig_feature.location.start)
        location_of_end=int(self.orig_feature.location.end)
        location_of_closest_start=int(self.new_feature.location.start)
        location_of_closest_end=int(self.new_feature.location.end)
        return
min(location_of_start,location_of_end,location_of_closest_start,location_of_closest_end)
    def getEndOfBoth(self):
        location_of_start=int(self.orig_feature.location.start)
        location_of_end=int(self.orig_feature.location.end)
        location_of_closest_start=int(self.new_feature.location.start)
        location_of_closest_end=int(self.new_feature.location.end)
        return
max(location_of_start,location_of_end,location_of_closest_start,location_of_closest_end)
    def getSubjEnd(self,feature):
        if feature.strand==1:
            return str(feature.qualifiers["sbjct_end"][0])
        if feature.strand==-1:
            return str(feature.qualifiers["sbjct_start"][0])
    def getSubjStart(self,feature):
        if feature.strand==1:
            return str(feature.qualifiers["sbjct_start"][0])
        if feature.strand==-1:
            return str(feature.qualifiers["sbjct_end"][0])
    def getShortestAA(self):
        return
min(self.orig_feature.qualifiers["ntlength"][0],self.new_feature.qualifiers["ntlength"][0])
    def getShortestNt(self):
        return
min(self.orig_feature.qualifiers["ntlength"][0],self.new_feature.qualifiers["ntlength"][0])
```
