# Supplementary material for: Frankia-Enriched Metagenomes from the Earliest Diverging Symbiotic Frankia Cluster: They Come in Teams
Source: Genome Biol Evol. 2019 Jul 19;11(8):2273–91. doi: 10.1093/gbe/evz153 (PMC6735867; doi:10.1093/gbe/evz153)
Supplement: evz153_Supplementary_Data [file evz153_supplementary_data.zip › Supplementary Table S1_new_new.docx]

**Supplementary Table S1. Inocula used in this study^1^.**

| **Inoculum** | **Source** | | **propagated in** | **Genome name(s)^2^** |
| --- | --- | --- | --- | --- |
|  | **Country** | **Plant species** |  |  |
| **Cj1** | Japan | *Coriaria japonica* | *Datisca glomerata* | Cj1_Dg_vc, Cj1_Dg_nod |
| **Cm1** | France | *Coriaria myrtifolia* | *Datisca glomerata* | Cm1_Dg_vc, Cm1_Dg_nod |
|  | France | *Coriaria myrtifolia* | *Coriaria myrtifolia* | Cm1_Cm_nod |
| **Cppng1** | Papua New Guinea | *Coriaria ruscifolia* | *Coriaria arborea* | Crpng1_Ca_nod |
| **Cv1** | California | *Ceanothus velutinus* | *Ceanothus thyrsiflorus* | Cv1_Ct_nod |
| **Dd1** | Alaska | *Dryas drummondii* | *Datisca glomerata* | Dd1_Dg_vc, Dd1_Dg_nod |
| **Dg1** | Pakistan | *Coriaria nepalensis* | *Datisca glomerata* | Dg1_Dg_vc (formerly Dg1), Dg1_Dg_nod1, Dg1_Dg_nod2 |
|  | Pakistan | *Coriaria nepalensis* | *Coriaria nepalensis* | Dg1_Cn_nod |
| **Dg2** | California | *Datisca glomerata* | *Datisca glomerata* | Dg2_Dg_vc (formerly Dg2) |

^1^Dg1 (Persson et al. 2011; Persson et al. 2015) and Dg2 (Nguyen et al. 2016) were published previously.

^2^for explanation of genome names, see Results and Discussion, first paragraph.
