# Supplementary figures and images for: Frankia-Enriched Metagenomes from the Earliest Diverging Symbiotic Frankia Cluster: They Come in Teams
Source: Genome Biol Evol. 2019 Jul 19;11(8):2273–91. doi: 10.1093/gbe/evz153 (PMC6735867; doi:10.1093/gbe/evz153)

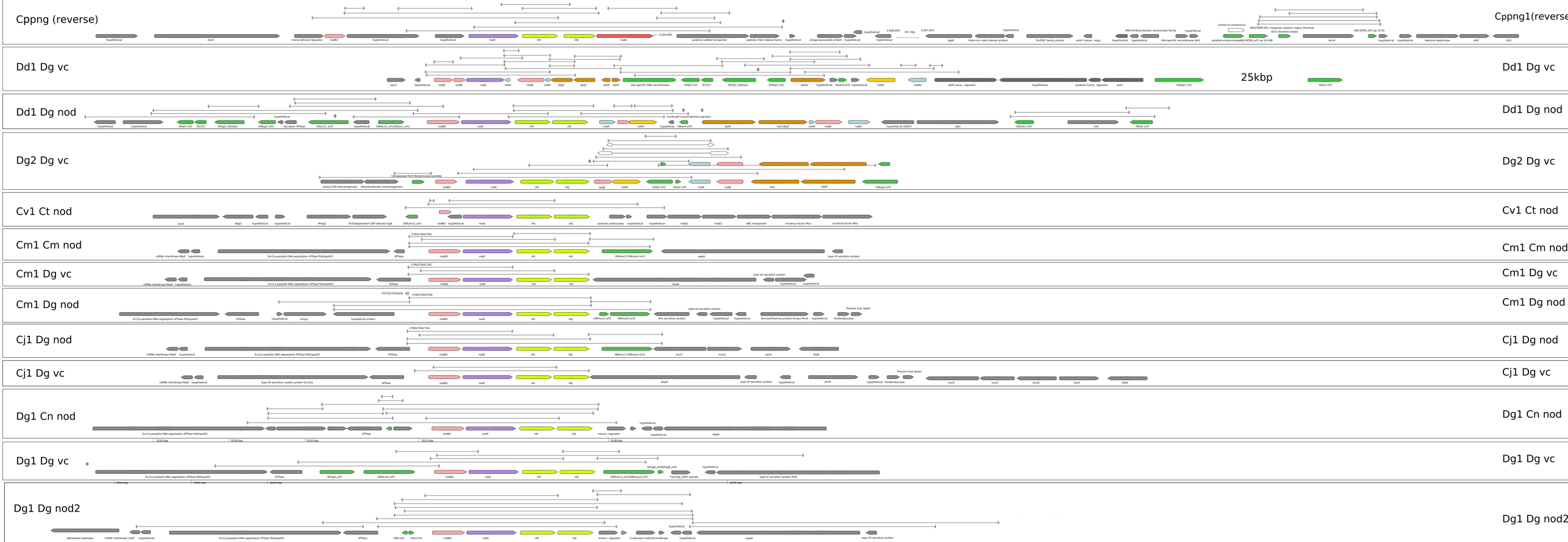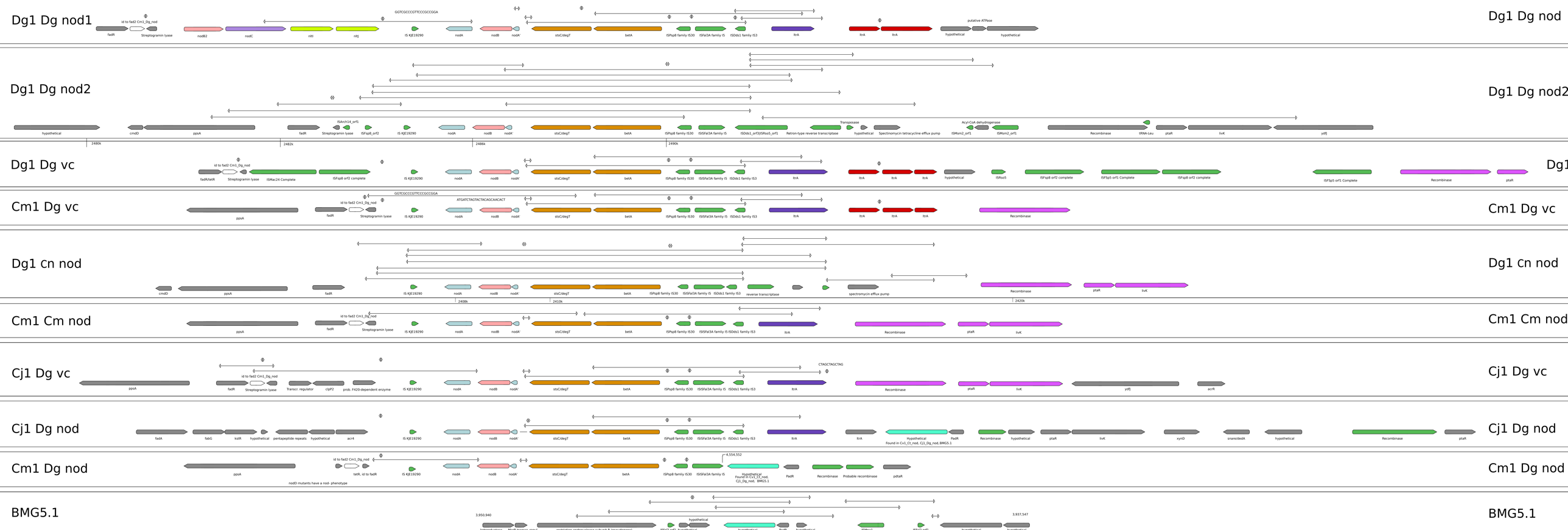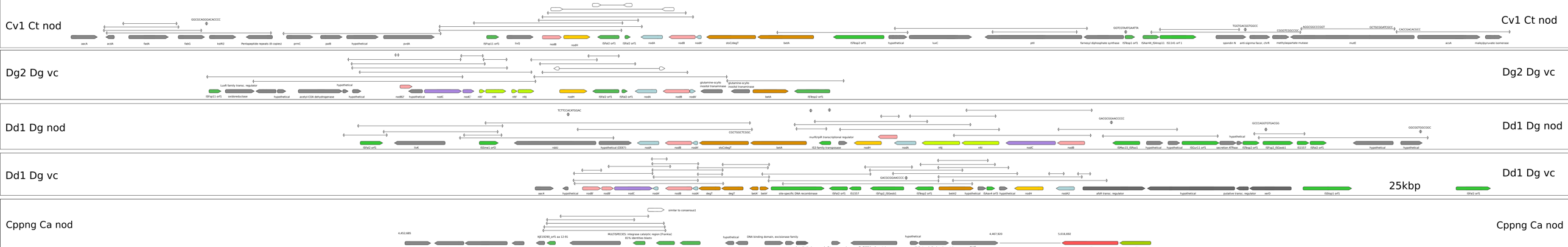

Supplement: evz153_Supplementary_Data [file evz153_supplementary_data.zip › Supplementary Fig S5new.pdf]
